# Multiple Molecular Pathways Are Influenced by Progranulin in a Neuronal Cell Model–A Parallel Omics Approach

**DOI:** 10.3389/fnins.2021.775391

**Published:** 2022-01-06

**Authors:** Babykumari P. Chitramuthu, Víctor R. Campos-García, Andrew Bateman

**Affiliations:** Division of Experimental Medicine, Faculty of Medicine and Health Sciences, McGill University, and Centre for Translational Biology, Metabolic Disorders and Complications, McGill University Health Centre Research Institute, Montréal, QC, Canada

**Keywords:** progranulin, FTD, CNS, RET, cell model, ALS, neurodegeneration, ceramides

## Abstract

Progranulin (PGRN) is critical in supporting a healthy CNS. Its haploinsufficiency results in frontotemporal dementia, while in experimental models of age-related neurodegenerative diseases, the targeted expression of PGRN greatly slows the onset of disease phenotypes. Nevertheless, much remains unclear about how PGRN affects its target cells. In previous studies we found that PGRN showed a remarkable ability to support the survival of NSC-34 motor neuron cells under conditions that would otherwise lead to their apoptosis. Here we used the same model to investigate other phenotypes of PGRN expression in NSC-34 cells. PGRN significantly influenced morphological differentiation, resulting in cells with enlarged cell bodies and extended projections. At a molecular level this correlated with pathways associated with the cytoskeleton and synaptic differentiation. Depletion of PGRN led to increased expression of several neurotrophic receptors, which may represent a homeostatic mechanism to compensate for loss of neurotrophic support from PGRN. The exception was RET, a neurotrophic tyrosine receptor kinase, which, when PGRN levels are high, shows increased expression and enhanced tyrosine phosphorylation. Other receptor tyrosine kinases also showed higher tyrosine phosphorylation when PGRN was elevated, suggesting a generalized enhancement of receptor activity. PGRN was found to bind to multiple plasma membrane proteins, including RET, as well as proteins in the ER/Golgi apparatus/lysosome pathway. Understanding how these various pathways contribute to PGRN action may provide routes toward improving neuroprotective therapies.

## Introduction

Neurodegenerative diseases of aging are a leading cause of death, disability, and dependency (Erkkinen et al., 2018). The importance of progranulin (PGRN) in maintaining the health of the aging brain is well established (Chitramuthu et al., 2017a). For example, a genome-wide association study identified *GRN* (the gene for PGRN) as the second most prominent locus conferring resilience against age-related deterioration of the cerebral cortex (Rhinn and Abeliovich, 2017). Clinically, heterozygous non-sense mutations of *GRN* that result in a loss of 50% of PGRN mRNA, cause FTD-GRN, a form of frontotemporal dementia that results from a progressive cortical atrophy and is, at present, almost always lethal (Baker et al., 2006; Cruts et al., 2006). FTD-GRN is a TDP-43 proteinopathy, that is, it is characterized by the accumulation of intracellular inclusions of ubiquitinated and phosphorylated C-terminal fragments of transactive response DNA binding protein 43 kDa (TAR DNA-binding protein 43 or TDP-43). Other important TDP-43 proteinopathies include amyotrophic lateral sclerosis (ALS) (Neumann et al., 2006), limbic-predominant age-related TDP-43 encephalopathy (Nelson et al., 2019), and traumatic encephalopathy (McKee et al., 2010). Missense mutations of *GRN* have been identified in diseases other than FTD, including ALS (Schymick et al., 2007; Sleegers et al., 2008; Cannon et al., 2013) and Alzheimer’s Disease (Brouwers et al., 2008), although these are rare. Moreover, some variants of the *GRN* gene act as modifiers of ALS progression, reducing the age of onset of the disease and shortening survival (Sleegers et al., 2008). Occasionally both alleles of *GRN* are mutated, leading to the complete loss of PGRN mRNA and resulting in a lysosomal storage disorder called neuronal ceroid lipofuscinosis (NCL) (Smith et al., 2012). GRN-related NCL is clinically distinct from FTD, but it is highly likely that milder defects in autophagy and lysosome function contribute to FTD-GRN pathology (Gotzl et al., 2014). The role of *GRN*-deficiency in FTD has prompted interest in the development of PGRN-based therapies by, for example, increasing PGRN levels in the brain (Arrant et al., 2017, 2018; Miyakawa et al., 2020), or promoting read-through of *GRN* non-sense mutations (Frew et al., 2020; Kuang et al., 2020).

Just as loss of PGRN is detrimental to brain health, enhanced expression of PGRN in the central nervous system (CNS) protected against the onset of several pathological phenotypes, at least in experimental models (Chitramuthu et al., 2017a). Thus, in mice, increased PGRN expression improved the NCL-like phenotype of PGRN knockout mice (Arrant et al., 2018), inhibited the onset of disease-like phenotypes in TDP-43-related ALS (Beel et al., 2018), as well as pathologies that are not related to TDP-43, such as genetic models of Alzheimer’s disease (Minami et al., 2014; Van Kampen and Kay, 2017), and chemically induced Parkinson’s disease (Van Kampen et al., 2014). In addition to a protective action in chronic neurodegenerative conditions, PGRN stimulated neuronal regeneration and reinnervation following acute peripheral nerve injury (Altmann et al., 2016) and exerted a protective action during acute cerebral ischemia (Kanazawa et al., 2015, 2019). Clearly, therefore, PGRN exerts a remarkably broad spectrum of protection across proteinopathy boundaries and in both chronic neurodegenerative diseases and acute neuronal injury.

Progranulin has multiple biological actions. It is neurotrophic (Van Damme et al., 2008; Ryan et al., 2009), it down-regulates microglial neuroinflammation (Martens et al., 2012), and it influences both lysosomal activity (Kao et al., 2017; Paushter et al., 2018; Elia et al., 2019) and autophagy (Chang et al., 2017; Elia et al., 2019; Doyle et al., 2021). Understanding how PGRN affects neuronal biology will provide insights into brain aging and resilience against neurodegeneration. Here we sought to employ a simple, readily accessible, cellular model to investigate how PGRN expression influences structural and molecular phenotypes. We have shown previously that cultures of the neuron-like cell line, NSC-34 modified to over-express PGRN, remained alive and in a differentiated state for at least 90 days in the complete absence of serum, whereas under the same serum-free conditions unmodified NSC-34 cells died within less than 10 days (Ryan et al., 2009). The ability of PGRN to support the survival of NSC-34 cells for months in the absence of any exogenous trophic support suggests that these cells are highly responsive to PGRN and may be a useful *in vitro* model for investigating cellular, molecular and biochemical aspects of PGRN action. NSC-34 cells were derived by the fusion of primary embryonic motor neurons with a neuroblastoma cell line (Cashman et al., 1992), and display many physiological properties of motor neurons, including the expression of neurofilament proteins, generation of action potentials, storage and release of acetylcholine, and induction of myotubular clustering of acetylcholine receptors (Cashman et al., 1992; Durham et al., 1993; Maier et al., 2013). They have, in addition, been widely used to study ALS-like TDP-43 neurotoxicity (Yang et al., 2010; Colombrita et al., 2012; Lu et al., 2012; Smethurst et al., 2016; Moujalled et al., 2017; Tian et al., 2017; Chen T. et al., 2018; Maurel et al., 2018; Salvatori et al., 2018; Hicks et al., 2020). PGRN is intimately involved in motor neuron function. In a semi-quantitative assessment motor neurons in the brain (pontine gray matter) and the spinal cord displayed the highest neuronal PGRN expression (Ryan et al., 2009) and PGRN is neurotrophic for motor neurons in primary tissue culture (Van Damme et al., 2008). In zebrafish, PGRN modulates the outgrowth and branching of primary motor neurons (Chitramuthu et al., 2010) and protects them from axonopathy caused by genes associated with motor neuron diseases including TDP-43 (Laird et al., 2010; Chitramuthu et al., 2017b), FUS (fused in sarcoma) (Chitramuthu et al., 2017b) and SMN1 (survival motor neuron 1) (Chitramuthu et al., 2010). It significantly delays the onset of TDP-43-dependent ALS-like phenotypes in mice (Beel et al., 2018). Here we have undertaken a series of parallel molecular analyses to identify how PGRN expression, or its depletion, affects the properties of NSC-34 motor neuron-like cells. The effect of PGRN insufficiency on the transcriptome of FTD-GRN brains has been reported at autopsy (Chen-Plotkin et al., 2008), or in Grn–/– mice (Chang et al., 2017). These analyzes, however, reflect late-stage events beyond the window of potential therapeutic intervention, and occur in a complex cellular environment where it is often difficult to assign observed effects to specific cells. Distinguishing effects that are the result of PGRN depletion itself from those that are secondary responses in reaction to the damage caused by PGRN depletion is not always clear. Moreover, the pathology of brains from Grn–/– mice differs from that of human FTD-GRN, and appears closer to NCL (Ahmed et al., 2010), while GRN+/− mice, that genocopy human FTD-GRN, show only mild phenotypes that are not fully representative of the human disease (Filiano et al., 2013). Cell-autonomous transcriptional analyses of PGRN insufficiency phenotypes have been performed using shRNA to diminish PGRN expression in primary human neural stem cells, where several pathways were affected (Rosen et al., 2011), whereas no transcriptional effect of GRN depletion was observed in induced pluripotent stem cell (iPSC)-derived neurons with GRN expression reduced by siRNA (Robin et al., 2020). The NSC-34 model allows the analysis of both PGRN insufficiency and elevated PGRN expression in a neuron-related cell that is highly dependent on intrinsic PGRN expression for survival and the maintenance of its neuronal phenotype.

## Materials and Methods

### Cell Culture, Transfection, and Validation

NSC-34 cells were used as previously described (Ryan et al., 2009) and were the gift of Dr. Heather Durham, (Montreal Neurological Institute, Montreal, QC). MDA-MB-468 cells were obtained from ATCC. Cells were grown in a 5% CO_2_ incubator at 37°C. NSC-34 cells were maintained in DMEM with 10% fetal bovine serum (FBS) (Cashman et al., 1992). MDA-MB-468 cells were maintained in DMEM with 10% FBS. Stable transfectants that express human PGRN (NSC-34 and MDA-MB-468 cells), were generated by transfection with human PGRN (pcDNA3-hPGRN) or pcDNA3 for empty vector control transfections. Cells were transfected using Lipofectamine (Invitrogen) and selected with G418 (400 μg/ml) for 4–6 weeks according to manufacturer’s instructions. To generate cells that stably express ShRNA-mPGRN, NSC-34 cells were transfected with shRNA constructs that were designed by OriGene Technologies using murine *Grn* specific 29 nucleotide stretches within the coding region. NSC-34 cells were seeded onto 6 well plates and then transfected with shRNA-mPGRN (pRS/shPGRN), or control vector (pRS vector alone, Origene Technologies), using Fugene (Roche) when the cultures were 80% confluent. Stable transfectants were selected by culturing in medium containing puromycin (7 μg/ml) (Invitrogen) for 6 weeks according to manufacturer’s instructions. To prevent phenotypic drift, stocks of the original transfectants were frozen in liquid N_2_ and reanimated at regular intervals.

### Reverse Transcription Polymerase Chain Reaction Analysis

Total RNA was isolated using Trizol reagent (Invitrogen). cDNA synthesis was performed with Revert-aid reverse transcriptase (Thermo Scientific). Human and mouse PGRN specific primer sets were designed to amplify species specific products. See Supplementary Table 1 for the sequences of the PCR primers. The polymerase chain reaction (PCR) program consisted of denaturation for 2 min at 94°C; 35 cycles at 94°C, 30 s; 55°C, 30 s; 72°C, 30 s; and a final extension of 5 min at 72°C. PCR was performed using Taq DNA Polymerase (Bio-Basic).

### Western Blot Analysis

Radioimmunoprecipitation assay (RIPA) buffer (Sigma) mixed with Complete Protease inhibitor Cocktail (Roche Applied Science, 1 tablet for 7 ml buffer) was used to lyse and extract proteins from NSC-34 cells. Protein lysates were incubated on ice for 5 min, briefly sonicated and centrifuged at 13,000 rpm for 15 min at 4°C. Supernatants were quantified for protein content and equal amounts were mixed with 2× sample buffer, boiled for 5 min and resolved on a 10% SDS-PAGE gel. Proteins on the gel were transferred onto a nitrocellulose membrane and blocked over-night with membrane blocking agent (GE Healthcare) or non-fat milk (Thermo Scientific) at 4°C. The blots were incubated with 1:250 anti-human PGRN polyclonal antibody (rabbit anti-human PGRN generated in our laboratory) or 1:10000 anti-human PGRN (R&D system) for 1 h in blocking buffer followed by extensive washing. After incubating with an anti-rabbit or anti–mouse IgG-horseradish peroxidase (HRP)-conjugated secondary antibody (diluted 1:4,000) at room temperature for 1 h and blots were visualized using enhanced chemiluminescence (GE Healthcare) according to the manufacturer’s instructions. Other antibodies used were 1:1000 anti-Ret (Cell Signaling Technology) and anti-phospho Ret (abcam), 1:2000 anti-EGFR, anti-phosphoEGFR, anti-ErbB2, anti-phosphoErbB2 (R&D systems). Protein loading controls were performed by stripping the membrane and probing with mouse monoclonal β-actin antibody (AC-40; Sigma) at a dilution of 1:1000.

### Morphometric Analysis

Cells were plated in 6 well plates at 7000 cells per well containing DMEM with 10% FBS. After 24 h, the media was replaced with DMEM containing 0% FBS, 1% FBS, or 10% FBS. The cultures were monitored for 25 days, and photographs were acquired using an inverted phase contrast microscope. The photographs were analyzed using ImageJ (Schindelin et al., 2012) by enabling the “Area” and “Shape” descriptors, and “Perimeter” and “Feret’s diameter” measurements (Rasband, 1977). Feret’s diameteris the longest distance between any two points along the selection boundary (Ferreira and Rasband, 2012), sometimes also called the “caliper length,” and is a measure of asymmetry. The neurite-like projections were measured using the NeuronJ plugin.^1^ This facilitates the tracing and quantification of neurite extension using the software ImageJ (Meijering et al., 2004; Meijering, 2010).

### Neurite Extension Assay

Cells were cultured in 12 well plates coated with nitrocellulose which was prepared by dissolving 5 cm^2^ of nitrocellulose (membrane 0.22 um, Bio basic) in 6 ml of methanol. Briefly 0.1 ml aliquots of this solution were rapidly spread over the surface of each well in 12 well plate and allowed to dry under a laminar flow hood as described in Lagenaur and Lemmon (1987). PGRN was applied in 3 μl droplets containing 100 μg/ml. After one minute, the droplets were removed gently using a pipette. The plates were then blocked by washing twice with 2 ml of culture media (DMEM with 10% FBS) for 10 min each. NSC- 34 cells were cultured on the nitrocellulose coated plates at the concentration of 6000 cells per ml in 2 ml medium supplemented with 10% FBS for 2 days. The neurite length was measured using ImageJ by tracing the neurite-like projections.

### Statistics for Neurite Extension and Morphometric Analyses

Statistical significance among experimental groups was determined by one-way ANOVA followed by Tukey’s Multiple Comparisons Test (*p* < 0.001-^***^, *p* < 0.01-^**^, *p* < 0.05-*) using GraphPad software (GraphPad Prism Software Inc., San Diego, CA). Error bars represent s.e.m.

### Transcriptomics Analysis

NSC-34/shPGRN, NSC-34/hPGRN, or control constructs were cultured in T75 flasks. They were sub-cultured in four replicates. NSC-34 cells (200,000) from each of 12 flasks were plated in T25 flasks with serum. After 24 h the media containing 10% serum was removed and replaced with media containing 5% serum. After a further 24 h the media was changed to one containing 1% serum. 24 h later cells were lysed in Trizol and total RNA was extracted. The RNA concentration was determined using a NanoDrop chip. cDNA synthesis was carried out using RevertAid First Strand cDNA Synthesis Kit according to the manufacturer’s protocol (Thermo Scientific). Following cDNA synthesis reverse transcription polymerase chain reaction (RT-PCR) was performed to confirm over/under expression of PGRN as indicated earlier. Reduced levels of mPGRN were noticed in all shPGRN constructs and expression of hPGRN was detected only in the over-expressors as expected. The over/under expression of PGRN levels were further confirmed by Western blot as mentioned previously.

The RNA quality/integrity was analyzed using a Nano ChiP Bioanalyzer. The gene expression profile was determined using Illumina Mouse Whole-Genome Expression BeadChips (MouseWG-6 v2.0, multiple 12 samples) at the McGill University and Genome Quebec Innovation Centre. The data replicates were validated by principal component analysis which confirmed that all 4 replicates from the same construct segregate together and separately from the other constructs. The lumi normalization followed by an empirical Bayesian, EB (Wright and Simon, 2003) algorithm was applied to the data using FlexArray 1.6.1 software (available at http://gqinnovationcenter.com/services/bioinformatics/flexarray/tutorials.aspx?l=e) to identify genes that were either significantly reduced or induced in the PGRN over/under expressing NSC-34 cells compared to controls. The Database for Annotation, Visualization, and Integrated Discovery (DAVID) v6.8 (Huang da et al., 2009b,a) was employed to provide tables of enriched Gene Ontology (GO) terms and predicted functional pathways based on the Kyoto Encyclopedia of Genes and Genomes (KEGG) database. Gene networks were analyzed using the Ingenuity Pathway analysis software (IPA). IPA is a web-based bioinformatics application that allows data analysis from microarray experiments by identifying related proteins within a pathway. 1.5-fold enrichment or depletion of transcript signal was used as the cut-off (Kramer et al., 2014). Additional functional assignments for individual genes were obtained manually by searching for their entries in Uniprot,^2^ Genecards^3^ and On-line Mendelian Inheritance in Man (OMIM^4^). Gene expression data was submitted to Gene Expression Omnibus (GEO) public repository, accession number: GSE185877.

### Lipidomic Analysis

Lipidomics and Proteomics mass spectrometric analyses were performed by the Proteomics Technology Platform, Research Institute of the McGill University Health Centre, Montreal (manager Lorne Taylor). NSC-34/shPGRN or NSC-34/hPGRN cells were cultured in T75 flasks in DMEM with 1% FBS. Cells were collected by trypsinization at about 70% confluency. Three independent extractions and mass spectrometric analyses were performed. Cells were centrifuged, resuspended in 1 ml and cell numbers were determined. Twenty million cells from each sample were collected by centrifugation at 3000 rpm for 5 min and incubated with 150 μl methanol and 500 μl Methyl tert-butyl ether (MTBE, Fisher Scientific) for 2 h on a rocker at room temperature. Phase-separation was performed by adding 125 μl of MS grade water (Fisher Scientific). After 10 min incubation at room temperature each sample was centrifuged at 3000 rpm for 10 min. The upper organic phase was collected, and the lower phase was re-extracted by adding methanol, MTBE, and water. Both fractions were combined, dried using a SpeedVac lyophilizer and were introduced into the TripleTOF™ 5600 System by direct infusion using the Nanospray III source and an Eksigent NanoLC Ultra and Autosampler at a flow rate of 1 μL/min. The data were analyzed using peak view Software and lipid identification and quantification was done in lipid view Software.

### Proteomics Analysis

NSC-34/ShPGRN, NSC-34-/hPGRN, or vector controls were cultured in T75 flasks in DMEM with 1% FBS until about 80% confluency. Cells were washed with PBS and lysed by homogenizing in solution containing 20 mM HEPES, 150 nM NaCl, and 0.320M sucrose. Supernatants were collected by centrifugation at 3000 rpm at 4°C for 10 min. Samples were normalized for input by cell numbers and subsequently by total peptides using the Scaffold program (see below) at the data analysis stage. For each sample, proteins were loaded onto a single stacking gel band to remove lipids, detergents and salts. The gel band was reduced with dithiothreitol, alkylated with iodoacetic acid and digested with trypsin. Extracted peptides were re-solubilized in 0.1% aqueous formic acid and loaded onto a Thermo Acclaim Pepmap (Thermo, 75 uM ID × 2 cm C18 3 uM beads) precolumn and then onto an Acclaim Pepmap Easyspray (Thermo, 75 uM × 15 cm with 2 uM C18 beads) analytical column separation using a Dionex Ultimate 3000 uHPLC at 230 nl/min with a gradient of 2–35% organic (0.1% formic acid in acetonitrile) over 3 h. Peptides were analyzed using a Thermo Orbitrap Fusion mass spectrometer operating at 120,000 resolution (FWHM in MS1) with HCD sequencing (15,000 resolution) at top speed for all peptides with a charge of 2+or greater. The raw data were converted into *.mgf format (Mascot generic format) for searching using the Mascot 2.6.2 search engine (Matrix Science) against mouse protein sequences (Uniprot 2018). The database search results were loaded onto Scaffold Q+ Scaffold_4.8.9 (Proteome Sciences) for statistical treatment and data visualization. Proteomics data was submitted to MassIVE public repository by participating in ProteomeXchange, accession number: MSV000088451. The Database for Annotation, Visualization, and Integrated Discovery (DAVID) v6.8 (Huang da et al., 2009b,a) was employed to analyze Gene Ontology (GO) as above using a 1.2-fold change cut-off and *p* < 0.05.

### Membrane Protein Interactomics of Progranulin by Ligand Receptor Capture

Ligand receptor capture (LRC) identifies the interactions of extracellular soluble proteins with membrane proteins in live cells by making use of a biotinylated trifunctional reagent TRICEPS (Frei et al., 2012). All reactions were performed according to the manufacturer’s protocols. 300 μg PGRN (R&D Systems), or insulin (Sigma-Aldrich) as the positive control, were coupled to 1.5 μl TRICEPS by an amide linkage. The second functional group of the TRICEPS reagent, a protected hydrazine, reacts with aldehydes on the glycan side chains of the target receptor proteins. As aldehydes are not typically present on the cell surface, they are first generated by mild sodium metaperiodate oxidation using 6 × 10^8^ NSC-34 cells. The cells were cultured in DMEM with 10% FBS and washed once with 1× PBS then in DMEM without serum for 30 min prior to cells collection. The LRC reaction was initiated by incubating mildly oxidized and washed living cells with 50 μl of the PGRN-TRICEPS conjugate, or 50 μl of Insulin-TRICEPS for 90 min at 4°C during which time PGRN-TRICEPS or insulin-TRICEPS reacts covalently through hydrazine coupling to the glycan chains of any cell surface membrane protein to which it binds. The cells were lysed, digested with trypsin and the TRICEPS labeled peptides recovered by biotin affinity using streptavidin beads. For mass spectrometry the streptavidin-purified peptides were de-glycosylated using PNGaseF, an enzyme which cuts between the proximal *N*-acetylglucosamine of the glycan side chain and the glycopeptide asparagine in the N-X-S/T glycosylation site motif (N stands for asparagine, X is any amino acid except proline and S/T is serine or threonine). The PNGase reaction deamidates the asparagine and modifies the N-X-S/T glycosylation motif to a D-X-S/T motif (where D is aspartic acid), the corresponding mass shift being used to identify peptides that were crosslinked by the PGRN or Insulin-TRICEPS conjugates. The TRICEPS labeled peptide recovery and the mass spectrometric analyses were performed by DualSystems Biotech. The samples were analyzed on a Thermo LTQ Orbitrap XL spectrometer. Peptide identifications were filtered to a false-discovery rate of ≤1% and quantified using an MS1-based label-free approach. For the MS1 quantification, the non-linear DYNAMICS Progenesis QI for proteomics was used. The experiment was done in triplicate. All six samples, PGRN and insulin control, showed an alignment score of >90%. Therefore, none of the six samples had to be excluded from statistical analysis. The Data is presented as a volcano plot where all the membrane proteins that crosslinked to either PGRN or the insulin control fall as statistical outliers on either arm of the plot. Positive detection of the insulin receptor control signal confirms that the TRICEPs reaction worked under the conditions used, and that there was no protein denaturation. Non-specific binding is accounted for as signals that are statistically indistinguishable between the insulin and progranulin arms of the experiment. Possible interactions among proteins identified in these assays was performed using the STRING program (Search Tool for the Retrieval of Interacting Genes/Proteins) (Szklarczyk et al., 2019).

### Phospho-Receptor Tyrosine Kinases Antibody Array

The proteome profiler mouse or human Phospho-Receptor Tyrosine Kinases (RTK) antibody array membranes (R&D Systems, Minneapolis, MN) were incubated with NSC-34 or MDA-MB-468 cell lysates and processed as per the manufacturer’s protocol using a Phosphotyrosine specific antibody. In brief, approximately 3 × 10^7^ cells of NSC-34/shPGRN cells expressing reduced PGRN, NSC-34/hPGRN cells overexpressing PGRN and WT NSC-34 control, MDA-MB-468 WT and PGRN expressing cells were cultured in serum free medium overnight. After incubation, cells were washed with ice-cold Dulbecco’s phosphate-buffered saline (DPBS) and lysed with lysis buffer containing aprotonin/leupeptin (10 μg/mL each) for 30 min, centrifuged at 13000 rpm for 5 min. Supernatants were collected and stored at –80 degrees A protein assay (BioRad Biotechnologies, Hercules, CA) was performed before incubating with the RTK membranes to normalize loadings.

## Results

### Progranulin Expression, Knockdown, and Over-Expression in NSC-34 Cells

NSC-34 vector control cells, and cells that either stably over-express human PGRN (NSC-34/hPGRN) or under-express murine PGRN (NSC-34/shPGRN) were generated (Supplementary Figure 1). PGRN levels were depleted by approximately 50% in NSC-34/shPGRN cells, a reduction equivalent to that associated with FTD-GRN (Supplementary Figures 1A,B). To confirm their neuron-like identity NSC-34 cells were stained for the motor neuron marker SMI32, which recognizes dephosphorylated epitopes of neurofilaments (Supplementary Figure 1C). Neither depletion nor over-expression of PGRN altered TDP-43 mRNA or protein expression (Supplementary Figure 1D).

### Progranulin Regulates NSC-34 Cell Neurite Outgrowth, Morphology, and Survival

**FIGURE 1:**
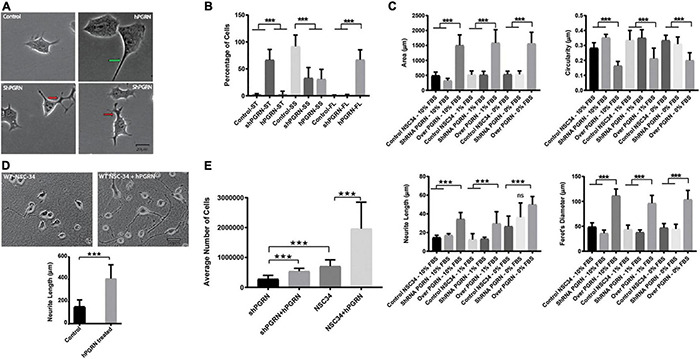
Morphological Profile and survival of NSC-34 cells. **(A)** Representative morphologies of NSC-34 cells cultured in 1% FBS for 48 h. PGRN over expressing cells show flattened enlarged cell bodies and longer neurite extensions (green arrow) than control cells. In the NSC-34/shPGRN cells some neurites condense to form well defined neurite-like extensions, but many are broad and tortuous with multiple branching points (red arrows), having the appearance of extensions that have failed to develop into organized neurite-like structures. These structures are rarely seen in NSC-34 PGRN over expressing cells or controls. **(B)** Cell morphologies were categorized into small cell bodies/short neurite extensions (SS), small cell bodies/tortuous neurite extensions (ST), and with flat cell bodies (FL). Most control cells show small cell bodies with short neurite extensions (control-SS), shPGRN cells show small cell bodies with tortuous neurite extensions (shPGRN-ST) and PGRN over expressing cells show flat cell bodies with long neurite extensions (hPGRN-FL) (189 control cells, 175 PGRN over expressing cells and 173 shPGRN cells from twenty fields were counted). **(C)** Morphometric data acquired in serum free, 1 and 10% serum over 8 days showed that high PGRN expression increased the cell body size, Feret’s diameter and Neurite length but decreased circularity under all conditions tested. **(D)** Exogenous PGRN is highly effective in supporting neurite outgrowth. WT NSC 34 cells were cultured on nitrocellulose coated plates pre-treated with 10% serum only or 10% serum supplemented with PGRN (1.33 nM). Cells were then cultured in 10% serum. After 2 days many cells plated on nitrocellulose enriched with human PGRN displayed long neurite-like extensions whereas cells that were plated on nitrocellulose without PGRN enrichment displayed fewer and shorter extensions (*N* = 20, 10× Fields per condition). **(E)** Cell number of NSC-34/shPGRN and NSC-34 control cells, with or without supplementation of the medium with PGRN (1.33 nM), was measured after an 8-day incubation in medium containing 1% FBS (*N* = 4) (*p* < 0.001-*** Error bars represent s.e.m.).

Progranulin expression had a strong impact on the shape and size of the cell body. At 48 h of culture, cell bodies of NSC-34/hPGRN have greater area and are flattened compared to the controls and NSC-34/shPGRN cells, which are typically small and circular (Figures 1A,B). Neurite length was greater in NSC-34/hPGRN cells than control or NSC-34/shPGRN cells when serum was present (10 or 1%). Extension in NSC-34/shPGRN often appeared to be poorly condensed and disorganized, which we refer to as “tortuous” (Figures 1A,B). Serum-deprivation stimulates differentiation in NSC-34 cells (Eggett et al., 2000) and, correspondingly, over an 8 day period in the absence of serum, all cells exhibited neurite extension with no significant difference detected in neurite length between NSC-34/hPGRN and NSC-34/shPGRN cells (Figure 1C). Extensive cell death occurs in the NSC-34/shPGRN cells after 8 days in serum free medium, at which point measurements were discontinued. PGRN is secreted but also localizes cytoplasmically, due, at least in part, to endocytosis into lysosomes (Hu et al., 2010; Petoukhov et al., 2013; Zhou et al., 2015, 2017). Neurons secrete PGRN in an activity dependent manner (Petoukhov et al., 2013). There are in addition multiple paracrine extracellular sources of PGRN including microglia (Ryan et al., 2009; Hu et al., 2010) and de-differentiated Schwann cells (Hyung et al., 2019). We therefore tested whether extracellular PGRN, mimicking the secreted protein, reproduces the morphological and survival phenotypes associated with endogenous PGRN expression. Exogenous PGRN added to the medium reproduced the effects of stable PGRN expression as WT NSC-34 cells displayed longer neurite outgrowth compared to cells cultured without PGRN on nitrocellulose coated plates pre-treated with PGRN in the presence of 10% serum (Figure 1D). Cell survival of NSC-34/shPGRN cells in 8-day serum-reduced culture decreased relative to WT NSC-34 cells as expected (Ryan et al., 2009), but adding PGRN to the medium restores NSC-34/shPGRN cell numbers to the levels of control NSC-34 cells (Figure 1E).

### Gene Expression Profiling

Given that the cells undergo active morphological differentiation within 2 days incubation in 1% serum with no loss of viability (Figure 1), all subsequent molecular analyzes were performed under these conditions. Gene expression microarrays were used to identify how the mRNA phenotype of NSC-34 cells responds to PGRN expression levels. The anticipated expression level of PGRN were confirmed in each of the NSC-34/shPGRN, control and NSC-34/hPGRN cell lines prior to the analysis (Supplementary Figure 2A). Genes showing statistically significant changes in expression were identified by FlexAarray analysis software. Principal component analysis confirmed that the data from each of NSC-34/shPGRN, NSC-34/hPGRN and vector controls cells segregates independently (Supplementary Figure 2B). Among genes that showed the greatest fold down-regulation in NSC-34/shPGRN cells compared to NSC-34/hPGRN cells (Table 1) were several associated with neurotransmission (dopamine decarboxylase, vesicular GABA transporter, alpha-2 adrenergic receptor), cytoskeletal organization (Stathmin 2, FGD2, SRGAP3), and RNA-binding proteins with roles in neuron development, survival, or congenital motor disorders (IGF2BP1, TSHZ1, GLE1).

**TABLE 1:** Top 20 down-regulated genes in NSC-34/shPGRN cells compared with NSC-34/hPGRN cells.

Gene symbol	Gene and implication	Fold change (down regulated)
HMCN1	Hemicentin-1	6.18
TTF1	Transcription termination factor 1	5.05
WFDC3	WAP four-disulfide core domain protein	5.01
SDSL	Serine dehydratase-like	3.74
STC1	stanniocalcin	3.69
IGF2BP1	Insulin-like growth factor 2 mRNA binding protein1 Axonal outgrowth 1	3.58
DDC	dopamine decarboxylase	3.44
STRA6	Stimulated by retinoic acid 6	3.08
STMN2	Stathmin 2	2.75
SLC32A1	Vesicular GABA transporter	2.65
FGD2	RhoGEF and PH domain containing 2 CDC42- specific exchange factor	2.63
6430517E21RIK	Similar to human fam5b	2.54
ADRA2A	Alpha-2-Adrenergic receptor	2.53
SRGAP3	Slit-Robo Rho GTPase-activating protein 3 Severe mental retardation	2.49
TSHZ1	Teashirt zinc finger homeobox 1 Motor neuron development, survival and function	2.49
CES1	Carboxylesterase 1	2.47
DKK1	Dickkopf-related protein 1	2.47
4933405K21RIK	GLE1A and GLE1B RNA export mediator Lethal congenital contracture syndrome and ALS	2.46
2310005C01RIK	neuronal nitric oxide synthase	2.43
EG433865	similar to spermidine/spermine N1-acetyltransferase	2.42

*Note the presence of genes associated with neurotransmission, cytoskeletal organization, and congenital neurological disorders. Among genes that showed the greatest fold down-regulation in NSC-34/shPGRN cells compared to NSC-34/hPGRN cells were several associated with neurotransmission (dopamine decarboxylase, vesicular GABA transporter, alpha-2 adrenergic receptor) (Azzouz et al., 2002; Bucheler et al., 2002; Johnson et al., 2003), cytoskeletal organization (Stathmin 2, FGD2, SRGAP3) (Endris et al., 2002; Budhachandra et al., 2008; Hayakawa et al., 2008; Carlson et al., 2011; Klim et al., 2019) and RNA-binding proteins with roles in neuron development, survival, or congenital motor disorders (IGF2BP1, TSHZ1, GLE1) (Nousiainen et al., 2008; Gaynes et al., 2015; Zhang et al., 2018; Chaimowicz et al., 2019).*

Ingenuity Pathway analysis identified potential PGRN-sensitive functional networks (Table 2). Among “biological function” networks PGRN knockdown was associated with neurological disease, behavior and nervous system development and function. The strong neurological disease-related signature in the downregulated data set of NSC-34/shPGRN cells implies that they exhibit disease-relevant molecular signals of neuronal disturbance, and this data set was, therefore, prioritized for further analysis. The top five IPA Associated Network Functions for PGRN knockdown cells versus PGRN over-expressors, based on a 1.5-fold cutoff difference for transcript expression, are given in Table 3. Details of each of the IPA Associated Network Functions are given in Supplementary Figure 3. Manual inspection of IPA network 1 predicts a potential sub-network associated with histone remodeling. Network 2 predicts two sub-networks associated with sterol metabolism and triglyceride metabolism, respectively. Network 4 predicts a sub-network associated with activation of RNA polymerase or RNA processing and Network 5 predicts two possible sub-networks associated with neuronal voltage-activated calcium channel ion-pore structure or regulation, and synaptic neurotransmitter vesicle formation/exocytosis.

**TABLE 2:** Ingenuity Pathway Analysis of Top Predicted Biological Functions associated with knockdown or over-expression of PGRN in NSC-34 cells.

Treatment groups	Top scoring IPA biological functions		
	Diseases and disorders	Molecular and cellular functions	Physiological system development and function
NSC-34/shPGRN vs vector control NSC-34- DOWN	Neurological Disease	Cell Morphology	Behavior
	*p* value 4.95E-06–2.20E-02	*p* value 1.34E-05–1.92E-02	*p* value 5.25E-06–2.04E-02
	# molecules 128	# molecules 121	# molecules 56
NSC-34/shPGRN vs NSC-34/hPGRN DOWN	Neurological Disease	Lipid Metabolism	Nervous System Development and Function
	*p* value 1.08E-05–1.99E-02	*p* value 1.67E-05–1.87E-02	*p* value 3.89E-06–1.94E-02
	# molecules 108	# molecules 74	# molecules 118
NSC-34/shPGRN vs vector control NSC-34- UP	Cardiovascular Disease	Cellular Growth and Proliferation	Embryonic Development
	*p* value 1.38E-06–1.23E-02	*p* value 1.11E-05–1.21E-02	*p* value 1.56E-06–1.16E-02
	# molecules 67	# molecules 148	# molecules 106
NSC-34/shPGRN vs NSC-34/hPGRN - UP	Cancer	Cellular Growth and Proliferation	Cardiovascular System Development and Function
	*p* value 1.98E-09–1.56E-02	*p* value 4.74E-07–1.50E-02	*p* value 2.09E-05–1.56E-02
	# molecules 172	# molecules 147	# molecules 63

*Note that molecules that decrease when PGRN is knocked down have a predicted association with neurological disease, behavior and nervous system development and function. Down regulated gene sets are indicated as DOWN and up-regulated as UP.*

**TABLE 3:** IPA Associated Network Functions predicted for molecules downregulated by more than 1.5-fold between NSC-34/shPGRN PGRN knockdown and PGRN overexpressing NSC-34/hPGRN cells.

Rank	ID Associated Network Functions	Score
1	Cell Cycle, Tissue Morphology, Cell-mediated Immune Response	47
2	Lipid Metabolism, Small Molecule Biochemistry, Vitamin and Mineral Metabolism	39
3	Infectious Disease, Cellular Development, Cellular Growth, and Proliferation	39
4	Cell Signaling, Gene Expression, Cell Cycle	38
5	Cell-To-Cell Signaling and Interaction, Nervous System Development and Function, Amino Acid Metabolism	34

**FIGURE 2:**
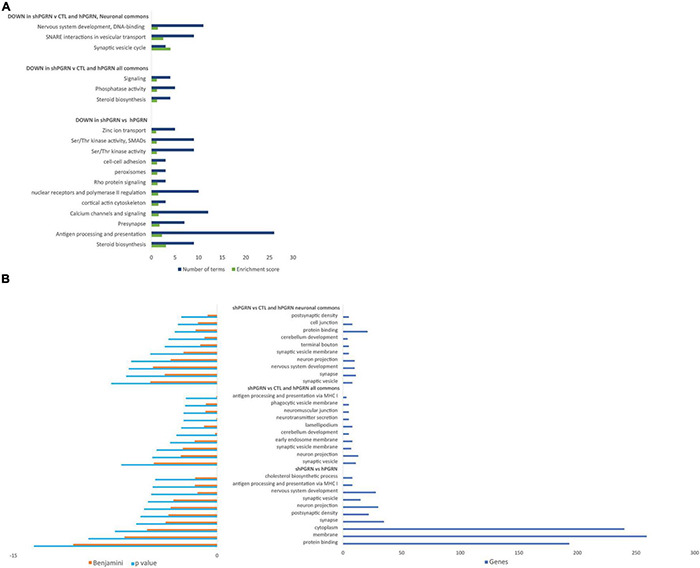
Gene ontology enrichment analyses for molecular signals that are downregulated in cells with reduced PGRN expression (NSC-34/shPGRN cells). **(A)** Functional Annotation Clusters (DAVID) with an enrichment score of one or greater for molecules downregulated in PGRN knockdown cells (shPGRN) are compared against: (i) PGRN overexpressing cells (DOWN in shPGRN vs hPGRN); (ii) The molecules downregulated in NSC-34/shPGRN cells relative to both NSC-34/hPGRN cells and Control cells (DOWN in shPGRN v [CTL and hPGRN all commons]) and molecules in the common set that have well defined neuronal functions (DOWN in shPGRN v [CTL and PGRN, neuronal commons]). See the text for details. Both GO and KEGG terms were included in the analysis and the clusters named based on a summary of its individual GO and KEGG terms. **(B)** Top ten GO terms downregulated in PGRN knockdown cells (shPGRN) versus groups (i–iii) as above. Statistical terms are expressed as -log_10_ and ranked by their *p* scores, and are shown together with their Benjamini scores, which take account of potential false discovery rates. The number of genes included in each GO term is given on the right.

Database for Annotation, Visualization, and Integrated Discovery Bioinformatics Resources were used to determine enrichment of GO terms and provide functional annotation clustering with groups, or clusters, of functionally related GO and KEGG terms ranked by an enrichment score (Huang da et al., 2009b,a). The DAVID analyses were stratified into: (i) signals that are downregulated in PGRN knockdown cells versus PGRN overexpressing cells; (ii) signals that are down regulated in PGRN knockdown cells with respect both to PGRN overexpressing cells and control cells (“commons”); (iii) the “neuronal commons,” that is genes extracted from the “common” subset that have a well-defined CNS function assessed by manually surveying their entries in the Uniprot, Genecards and OMIM databases. 657 genes in total go down in the [shPGRN vs hPGRN] gene set. The common gene set [shPGRN vs CTL/hPGRN] contains 264 molecules (40% of all down-regulated genes in the [shPGRN vs hPGRN] gene set). There are 55 genes in the “neuronal commons” gene subset, representing one fifth (20.8%) of the common gene set. Predicted functional annotation clustering is given in Figure 2A for clusters with an enrichment score of one or above. The top 10 most enriched individual GO-terms are given in Figure 2B. Both the cluster analysis and individual GO-terms indicate effects on synaptic function in the NSC-34/shPGRN cells (compare with IPA network 5, Supplementary Figure 3), associated, in particular, with synaptic vesicle cycling and SNARE interactions in vesicular transport and neuron projection. Functional annotation clustering (Figure 2A) and the list of top individual GO terms (Figure 2B) also show a strong signal for sterol biosynthetic pathways (compare with IPA network 2, Supplementary Figure 3).

The top GO-terms and annotation clusters that are upregulated in NSC-34/shPGRN cells relative to both control cells and PGRN over-expressing cells (commons-up) are summarized in Supplementary Table 2. This revealed several signals for neuron-related terms, but, rather than being centered on synaptic structural functions, as was observed among the down-regulated data set (Figure 2), the up-regulated terms are related to semaphorin signaling and extracellular axon guidance pathways. The GO term “membrane” was enriched in both the down-regulated (Figure 2B) and up-regulated transcript data sets (Supplementary Table 2). Among the molecules included in the two “membrane” GO terms (up and down regulated), are many with receptor-like properties (Table 4). Substantially more signals for receptor tyrosine kinase transcripts (AXL, EPHB4, NGFR, and PDGFRB) are up-regulated in NSC-34/shPGRN cells compared to those that are down-regulated (RET). Similarly signals for molecules in the semaphorin-signaling pathway (NRP1, NRP2, PLXNA1, SEMA7A, SEMA6B), Notch-related receptors (NOTCH1 and NOTCH4), and receptors- in the Wnt-signaling pathway (FZD3, FZD7, LGR6) were up-regulated when PGRN was depleted. In comparison, the number of differentially regulated G-protein linked receptor mRNAs in either the up- or the down-regulated groups is similar (5 vs 6). Receptor-like proteins associated with the immune response were down-regulated in NSC-34/shPGRN cells (CD109, CD180, CD40, Fc receptor IgA IgM, CSF2RA, TREML1, and TNFSF8). Although PGRN is a powerful survival agent in NSC-34 cells we did not detect any signals associated with apoptosis or its inhibition in NSC-34/shPGRN cells. This is, however, unsurprising as the conditions chosen for the gene expression analyses were not associated with extensive cell death.

**TABLE 4:** A comparison of membrane proteins identified within the GO term “membrane” with receptor-like properties that are either down regulated or up regulated in shPGRN cells relative to both control cells and PGRN over-expressing cells.

	Down regulated		Up regulated

**Symbol**	**Gene name**	**Symbol**	**Gene name**
	**RECEPTOR TYROSINE KINASES**		**RECEPTOR TYROSINE KINASES**
RET	Ret proto-oncogene (Ret)	AXL	AXL receptor tyrosine kinase (Axl)
	**G-PROTEIN COUPLED**	EPHB4	EPHB4 Epr receptor B4 (Ephb4)
GPR19	G protein-coupled receptor 19 (Gpr19)	NGFR	Nerve growth factor receptor (Ngfr)
GPR85	G protein-coupled receptor 85 (Gpr85)	PDGFRB	Platelet derived growth factor receptor, beta polypeptide (Pdgfrb)
OPRL1	Opioid receptor-like 1 (Oprl1)		**G-PROTEIN COUPLED**
PTGER3	Prostaglandin E receptor 3 (subtype EP3) (Ptger3)	ADRA1D	Adrenenrgic receptor, alpha 1d (adra1d)
SSTR2	Somatostatin receptor 2 (Sstr2)	GPR82	G protein-coupled receptor 82 (Gpr82)
	**WNT-SIGNALING**	GRM1	Glutamate receptor, metabotropic 1 (Grm1)
	Nil	PTGIR	Prostaglandin 1 receptor (IP) (Ptgir)
	**SEMAPHORIN-SIGNALING**	P2RY6	Pyrimidinergic receptor P2Y, G-protein coupled 6 (P2ry6)
	Nil	TSHR	Thyroid stimulating hormone receptor (Tshr)
	**NOTCH RECEPTORS**		**WNT-SIGNALING**
	Nil	FZD3	Frizzled class receptor 3 (Fzd3)
	**IMMUNE RECEPTORS**	FZD7	Frizzled class receptor 7 (Fzd 7)
CD109	CD109 antigen (Cd109)	LGR6	Leucine-rich repeat-containing G protein-coupled receptor 6 (Lgr6)
CD180	CD180 antigen (Cd180)		**SEMAPHORIN-SIGNALING**
CD40	CD40 antigen (Cd40)	NRP1	Neuropilin 1 (Nrp1)
FCAMR	FC receptor, IgA, IgM, high affinity (Fcamr)	NRP2	Neuropilin 2 (Nrp2)
CSF2RA	Colony stimulating factor 2 receptor, alpha, low-affinity (granulocyte-macrophage)	PLXNA1	Plexin A1 (Plxna1)
TREML1	Triggering receptor expressed on myeloid cells-like 1 (Treml1)		**NOTCH RECEPTORS**
TNFSF8	Tumor necrosis factor (ligand) superfamily, member 8 (Tnfsf8)	NOTCH1	Notch 1 (Notch1)
	**OTHERS**	NOTCH4	Notch 4 (Notch4)
GABRB3	Gamma-aminobutyric acid (GABA) A receptor, subunit beta 3 (Gabrb3)		**IMMUNE RECEPTORS**
SORCS3	Sortilin-related VPS10 domain containing receptor 3 (Sorcs3)	CD200	CD200 antigen (cd200)
VLDLR	Very low density lipoprotein receptor (Vldlr)	NCR1	Natural cytotoxicity triggering receptor 1 (Ncr1)
			**OTHERS**
		RELL1	RELT-like 1 (Rell1)
		EFNA4	Ephrin A4 (Efna4)
		FGFRL1	Fibroblast growth factor receptor-like 1 (Fgfrl1)
		GFRA3	Glial cell line derived neurotrophic factor family receptor alpha 3
		IGF2R	Insulin-like growth factor 2 receptor (Igf2r)
		SHH	Sonic hedgehog (Shh)

### Validation of Gene Expression

**FIGURE 3:**
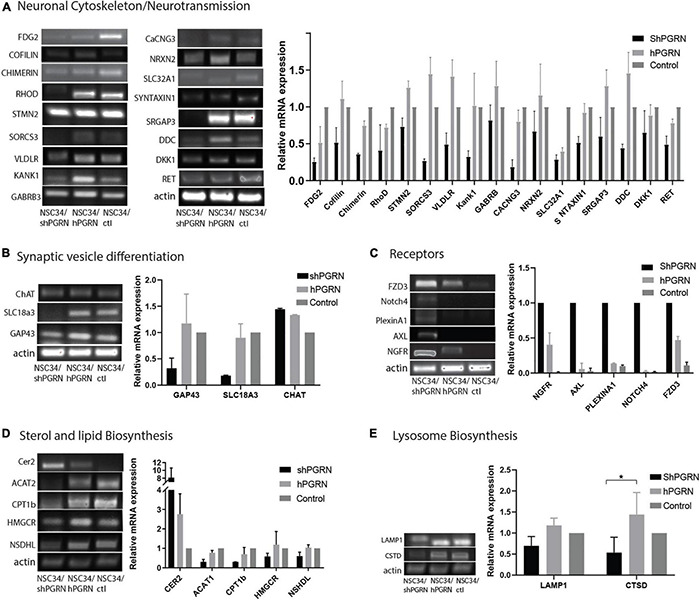
RT-PCR validation of gene expression patterns. **(A)** Selected molecules associated with neuronal cytoskeleton and synaptic transmission are sensitive to PGRN expression levels and are downregulated when PGRN levels are reduced. **(B)** RNA markers of axonal growth cone development (GAP-43) and cholinergic vesicle differentiation (SLC18A3) are regulated by PGRN expression but not the enzyme Choline acetyl transferase. **(C)** Transcripts for multiple neurotrophic receptors are upregulated upon PGRN depletion. **(D)** Pathways of sterol and lipid biosynthesis are sensitive to PGRN. **(E)** There is no consistent change in the expression of mRNA for lysosomal markers (*p* < 0.05-*, Error bars represent s.e.m.). All values are normalized to vector control cell levels which are shown as equal to one (*N* = 2± s/e/m for A–D and *N* = 3 for 3E *p* < 0.05.) except panel **(C)** which is expressed relative to NSC-34/shPGRN in order to avoid division by zero.

The reduced expression of genes involved in the neuronal cytoskeleton and neurotransmission in cells with depleted PGRN expression was confirmed by RT-PCR (Figure 3A). This is consistent with a role for PGRN expression in the maintenance of a differentiated state in NSC-34 cells (Figure 3A). Interestingly, there was often little difference in mRNA expression between control and NSC-34/hPGRN cells. We tested specifically for active growth cone formation using growth associated protein 43 (GAP-43, also called neuromodulin) (Maier et al., 2013), and, for evidence of cholinergic differentiation using vesicular acetylcholine transporter (SLC18A3) and choline acetyl transferase (ChAT) as markers (Figure 3B). Expression of GAP-43, and SLC18A3 cells were unchanged between control and NSC-34/hPGRN cells but were decreased in NSC-34/shPGRN, further supporting a role for PGRN in supporting neuron-like structural differentiation in NSC-34 cells. In contrast ChAT mRNA levels, were unaffected by PGRN depletion. Increased expression of neurotrophic receptor transcripts in NSC-34/shPGRN cells was confirmed, with strong signals observed for NGFR (nerve growth factor receptor) and FZD3 (Frizzled 3, a wnt receptor) (Figure 3C). A concerted change in genes associated with lipid metabolism was confirmed (Figure 3D) with most genes downregulated in NSC-34/shPGRN cells compared to control or NSC-34/hPGRN cells as predicted. Not all lipid metabolism genes were downregulated, however, as mRNA for ceramide synthase 2 (CER2) was strongly upregulated.

Neither IPA nor DAVID predicted a strong lysosomal response to PGRN depletion even though deletion of PGRN results in altered expression of lysosomal genes and proteins in other cell lines and *Grn* knockout mice (Tanaka et al., 2017; Huang et al., 2020). To evaluate whether PGRN depletion alters lysosome gene expression in NSC-34 cells we measured the relative mRNA levels of the lysosome proteins lysosomal associated membrane protein 1 (LAMP1) and cathepsin D (CTSD). There was no indication of increased expression of these genes in NSC-34/shPGRN cells (Figure 3E), and an apparent decrease in expression of cathepsin D, which implies a lack of extensive lysosome biogenesis upon partial PGRN depletion in this model.

### Lipid Groups Are Differentially Regulated in Progranulin Knockdown NSC-34 Cells

**FIGURE 4:**
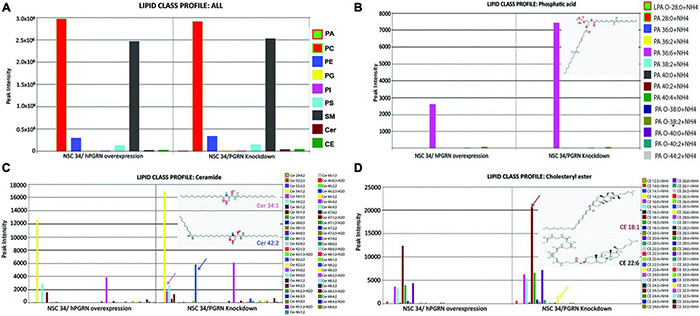
Lipidomic analyses of NSC-34 cells that stably express hPGRN compared to PGRN knockdown cells showing overall stability of the major lipid classes between the two treatments. **(A)** There are no differences in quantitatively dominant lipid classes in NSC-34/shPGRN versus NSC-34/hPGRN cells. PGRN depletion is associated with increases in quantitatively minor lipid components including specific members of **(B)** the Phosphatidic acids (insert showing the structure of Dimyristoyl phosphatidic acid), **(C)** the ceramides (Cer 34:1, Cer 42:2), and **(D)** cholesterol esters (CE18:1,CE22:6) (PA, Phosphatidic acids; PC, Glycerophosphocholines; PE, Glycerophosphoethanolamines; PG, Glycerophosphoglycerols; PI, Glycerophosphoinositols; PS, Glycerophosphoserines; SM, Sphingomeylins; Cer, Ceramides; CE, cholesterol esters).

Since the transcriptomic data suggested that lipid metabolic pathways are sensitive to PGRN depletion, we undertook mass spectrometric lipidomic analyses on NSC-34/shPGRN and NSC-34/hPGRN cells. Principal component analysis confirmed that all 3 replicates from the same cell construct segregate separately from the other constructs (Supplementary Figure 4). High or low PGRN expression had no effect on the levels of the quantitatively dominant lipid groups (Figure 4A). In contrast, three lipid classes that are quantitatively less abundant, phosphatidic acid, ceramides and cholesteryl ester show upregulation of specific lipid members between high and low PGRN expressors (Figures 4B–D).

### Influence of Progranulin on Protein Expression

**FIGURE 5:**
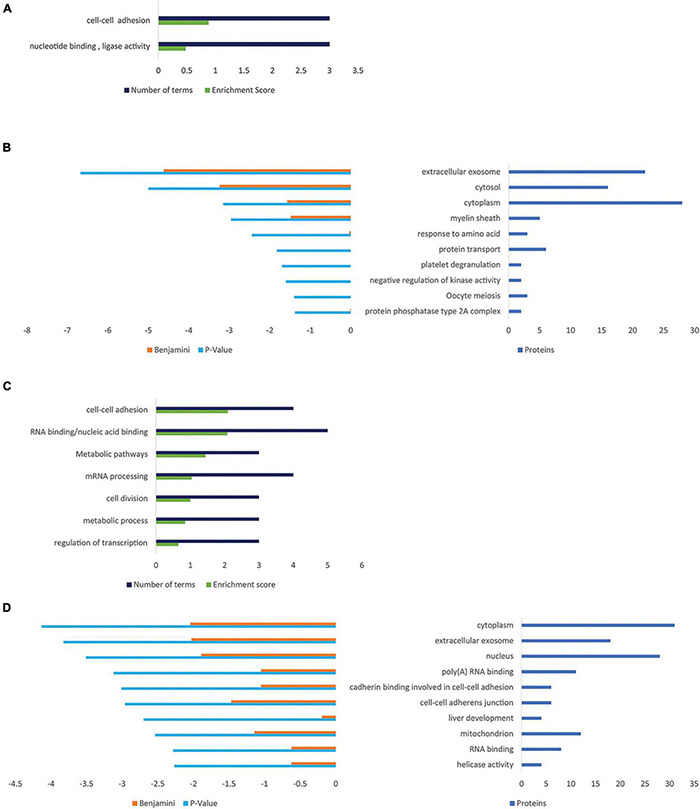
Gene ontology enrichment analyses for proteins that are regulated by PGRN expression in NSC-34 cells. **(A)** Functional Annotation Clusters (DAVID) enriched for proteins overexpressed in NSC-34/hPGRN cells compared to NSC-34/shPGRN cells. Both GO and KEGG terms were included in the analysis. **(B)** Top ten GO terms upregulated in NSC-34/hPGRN cells compared to NSC-34/shPGRN cells. **(C)** Functional Annotation Clusters (DAVID) enriched for proteins overexpressed in NSC-34/shPGRN cells compared to NSC-34/hPGRN cells. Both GO and KEGG terms were included in the analysis. **(D)** Top ten GO terms upregulated in NSC-34/shPGRN cells compared to NSC-34/hPGRN cells. Statistical terms are expressed as –log_10_ and ranked by their *p* scores, and are shown together with their Benjamini scores. The number of genes included in each GO term is given on the right.

The influence of PGRN expression on cellular protein composition was determined comparing NSC-34/hPGRN, NSC-34/shPGRN cells, and control cells, respectively (Supplementary Figure 5). The largest variability was detected between PGRN enriched NSC-34/hPGRN cells and NSC-34/shPGRN knockdown cells. Enrichment of GO and Kegg terms identified functional annotation clusters associated with cell adhesion, and metabolism (Figures 5A,B). Functional annotation clusters (Figure 5C) and enriched GO terms (Figure 5D) for proteins that were overexpressed in NSC-34/shPGRN cells were linked to cell adhesion, RNA binding, RNA processing and metabolism.

### Progranulin Binds to Multiple Membrane Proteins Both on the Cell Surface and in the Endoplasmic Reticulum/Golgi/Lysosome Network

**FIGURE 6:**
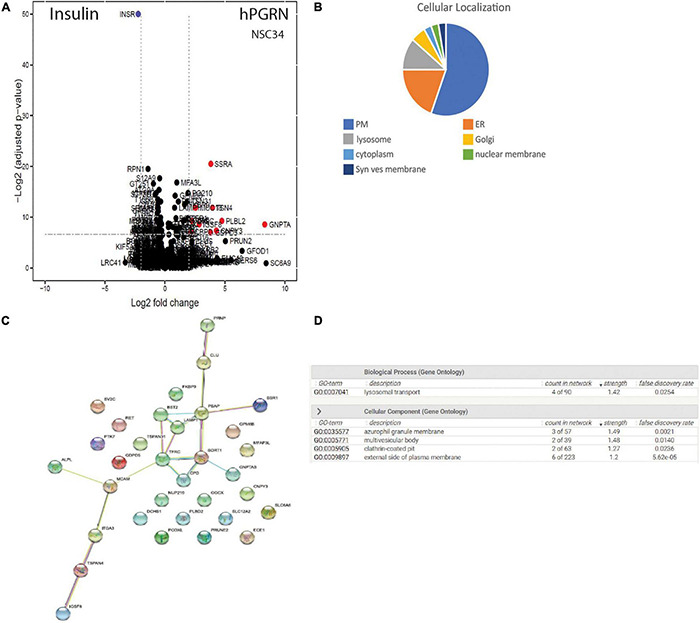
PGRN binds to multiple membrane proteins. **(A)** CaptiRec volcano plot to compare enriched proteins analyzed by ligand-receptor capture in the Insulin and PGRN samples. The data is shown as the log2 fold change (logFC) of protein level against the adjusted *p* value. **(B)** Cellular localization of putative PGRN-binding proteins identified by ligand receptor capture. Most of these proteins are associated with the plasma membrane, the endoplasmic reticulum or lysosomes (PM, plasma membrane: ER endoplasmic reticulum; Syn. Ves. Membrane, synaptic vesicle membrane). **(C)** A STRING analysis of the data set (log2 fold binding enrichment > 0.6 equivalent to approximately 1.5-fold change, and *p* value < 0.02) generated an interaction network of candidate lysosomal PGRN binding proteins that includes sortilin (SORT1), prosaposin (PSAP), and LAMP1. **(D)** Gene ontology (GO) analysis of biological processes in this data set gave the highest scores for lysosomal transport.

As the observed PGRN-dependent phenotypes in NSC-34 cells are reproduced by extracellular PGRN (Figures 1D,E), implying an action on extracellular membrane proteins (Chitramuthu et al., 2017a; Paushter et al., 2018), we therefore extended the proteomic analysis to probe specifically for potential PGRN membrane binding proteins using a LRC proteomic strategy (Frei et al., 2012, 2013; Figure 6A). In total 1746 glycopeptides were identified and quantified. Of these, the cut-off criterion employed for inclusion as putative PGRN-binders was a log2 fold binding enrichment > 0.6 (which is equivalent to an approximately 1.5-fold change in absolute values), and a *p* value < 0.02 (Supplementary Table 3). This yields 33 proteins. Most of the putative PGRN-binding proteins are associated with the plasma membrane, ER, or lysosome membranes (Figure 6B). Applying STRING analysis to this data set generated a potential interaction network of candidate lysosomal PGRN binding proteins that includes sortilin (SORT1, log2 fold enrichment 1.00 or 2 in natural numbers) and prosaposin (PSAP, log2 enrichment 0.716 or 1.64 in natural numbers) (Figure 6C). GO analysis of biological processes gave the highest score for lysosomal transport (Figure 6D). The presence of ER/Golgi/lysosome proteins is likely to be due to their exposure on the cell surface rather than uptake of labeled PGRN into the cells as the binding experiments were all conducted at 4°C. The highest scoring plasma membrane protein (Supplementary Table 3) was the tetraspanin TSPAN4 (log2 fold enhancement 3.96 or 15.5-fold in natural numbers). A second tetraspanin, TSPAN31 (log2 fold enhancement 1.86 or 3.6-fold in natural numbers), was also captured. Two members of the RTK-family, RET (log2 fold enrichment 1.25 or 2.4-fold in natural numbers), which is the kinase subunit of the glial cell-derived nerve growth factor (GDNF) receptor, and PTK7 (protein tyrosine kinase 7, log2 fold enrichment 0.942875 or 1.92-fold in natural numbers), which is an inactive RTK homolog. In the insulin-binding control arm, multiple peptides for insulin (INSR) were identified (log2 fold enrichment 2.23 or 4.69 in natural values).

### Progranulin Increases Activity of Different Receptor Tyrosine Kinases in Different Cell Types

**FIGURE 7:**
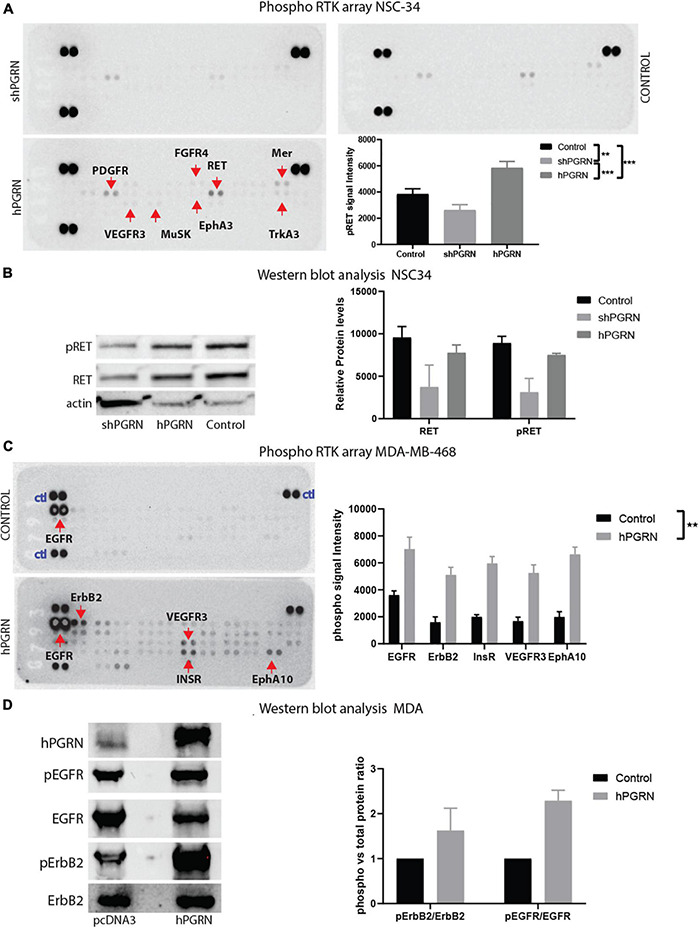
PGRN expression levels influence RTK phosphorylation. **(A)** An RTK phosphotyrosine array shows increased tyrosine phosphorylation of multiple growth factor receptors including RET between NSC-34/shPGRN and NSC-43/hPGRN cells. **(B)** This was confirmed for RET, by Western blot analysis, while **(C,D)** in MDA-MD-468 cells PGRN expression increased tyrosine phosphorylation of a different set of receptors including EGFR, ErbB2, Insulin-R, VEGFR3, EphA10 (Exposure time is one minute except for ErbB2, which is approximately 10 min) (*p* < 0.001-***, *p* < 0.01-**, Error bars represent s.e.m.).

Receptor phosphotyrosine antibody arrays were employed to screen for RTKs that are sensitive to PGRN expression levels (Figure 7A). RET, PDGFR-alpha (Platelet derived growth factor -alpha), Mer, and weakly, TrkA (high affinity nerve growth factor receptor), MusK (Muscle-Specific Kinase), and EphA3 (EPH Receptor A3) were more highly tyrosine phosphorylated in NSC-34/hPGRN cells than in vector controls or NSC-34/shPGRN knockdown cells (Figure 7B). Other reports, using the same phosphotyrosine receptor array screening technique, but analyzing non-neuronal cells, revealed that EphA2 was the likely RTK target for PGRN (Neill et al., 2016). Given that EphA3 phosphorylation is only weakly enhanced by PGRN expression in NSC-343 cells, we tested PGRN in MDA-MD-468 mammary epithelial cells and detected phosphorylation of the insulin receptor, the epidermal growth factor (EGF) receptor, and ErbB2 (an EGF-receptor family member), as well as members of the EphA family of receptors (Figure 7A). PGRN expression, therefore, activates different sets of tyrosine kinase receptors in different cell types implying cell context-dependent signaling. The antibody array results were validated by Western blot analyses using phospho-receptor specific antibodies (Figures 7C,D). RET expression is greatly reduced in NSC-34/shPGRN cells, suggesting that at least some of the differences observed in receptor RET tyrosine phosphorylation reflect differential expression of the RET protein, as was also predicted from the mRNA microarray analyses.

## Discussion

Motor neurons express PGRN at very high levels (Ryan et al., 2009) and are distinguished by the size of their cell bodies and the length of their axons. Based on results for NSC-34, PGRN may contribute to maintaining their morphology, as high PGRN expression clearly led to cell body enlargement and promoted axon extension, whereas PGRN depleted cells were unable to enlarge, and extensions were often unusually branched and poorly condensed. This, together with the previously reported dependency of NSC-34 cells upon PGRN for survival in the absence of trophic support from serum (Ryan et al., 2009), implies a marked trophic effect for PGRN on NSC-34 cells.

The morphological response to PGRN expression correlated well with molecular phenotypes that included mRNA pathways associated with the regulation of the cytoskeleton. This was further supported by changes in cell adhesion pathways in the proteome of NSC-34/hPGRN and NSC-34/shPGRN cells. PGRN depletion also impacted pathways associated with synaptic structure and function with presynaptic pathways particularly sensitive to PGRN depletion. These observations in NSC-34 cells are consistent with *in vivo* observations, where PGRN exerts complex roles in maintaining synaptic structure and connections (Tapia et al., 2011; Petkau et al., 2012; Petoukhov et al., 2013; Uesaka et al., 2018). Importantly, synaptic defects precede the onset of overt neuropathology in *Grn* knockout mice (Petkau et al., 2012), but it is unclear how PGRN influences synaptic structure. Synaptic pruning by microglia (Lui et al., 2016), and sortilin-mediated PGRN synaptic strengthening between climbing fibers and Purkinje cells during development (Uesaka et al., 2018), have been reported. The results from NSC-34 cells suggest that additional cell-autonomous mechanisms may also be involved.

Progranulin depletion in NSC-34 cells altered the expression of genes involved in lipid metabolism, although there was no global change in the levels of the quantitatively dominant lipid classes such as di- and triacyl glycerides and phosphatidylserines. Alterations in these lipid classes have, however, been observed in *Grn–/–* and *Grn*+/− mouse brains, probably as the result of lysosomal abnormalities (Evers et al., 2017). In contrast, lipids in the less abundant cholesterol ester, ceramide and phosphatidic acid families were elevated in PGRN-depleted NSC-34 cells. Increased ceramide expression is possibly due, in part, to increased CER2 (ceramide synthase 2) expression. There is increasing evidence, however, that PGRN intervenes at several key points in ceramide and sphingolipid metabolism. It modulates the activity of at least two enzymes involved in sphingolipid metabolism, glucocerebrosidase (GBA) (Jian et al., 2016a,b; Arrant et al., 2019; Valdez et al., 2020), and hexosaminidase A (Chen Y. et al., 2018). In addition, it binds to prosaposin, and regulates its trafficking and processing to saponins (Zhou et al., 2017; Valdez et al., 2020) which are non-enzymatic lysosomal proteins that promote sphingolipid catabolism. Ceramides play important roles in neurodegeneration (Cutler et al., 2004), and it will be important to determine to what extent they may contribute to the survival and morphological defects of NSC-34/shPGRN cells.

Although PGRN exercises a strong cell autonomous trophic action in NSC-34 cells, it remains unclear how this is exerted. Since extracellular PGRN replicated these actions, binding to membrane proteins is probably required, and we chose, therefore, to further investigate the properties of PGRN binding to NSC-34 cell membrane proteins. Several putative PGRN receptors and binding proteins have been proposed. Among them are heparan sulfate proteoglycans (Gonzalez et al., 2003; Yip et al., 2014), EphA2 (Neill et al., 2016), Tumor necrosis factor receptor 2 (Tang et al., 2011), Toll-like receptor-9 (Park et al., 2011), notch receptors (Altmann et al., 2016), components of lysosomal trafficking such as sortilin (Hu et al., 2010) and prosaposin (Zhou et al., 2015), and lysosomal enzymes involved in lipid and protein metabolism (Paushter et al., 2018). Reconciling these diverse binding proteins into an integrated model of PGRN action is challenging, particularly since PGRN may interact with more than one membrane protein at the same time. We employed LRC, a membrane interactomic strategy to obtain an unbiased survey of membrane-bound PGRN binding proteins in live NSC-34 cells, and thereby probe the nature and range of PGRN binding to membrane proteins. PGRN captured multiple membrane proteins (Figure 6 and Supplementary Table 3), of which several (12 of 33) have equal or stronger enrichment for PGRN binding than the classic receptor ligand insulin had for its receptor, although with lower *p* values. GNPTA (N-acetylglucosamine-1-phosphotransferase subunits alpha/beta), for example, has a log2 fold enrichment of 8.31 (or 317 in natural numbers), compared to a log2 fold enrichment of 2.23 for the insulin receptor. The lower *p* values in the PGRN versus the insulin arm are probably due to the capture of fewer unique peptides per protein than for the insulin receptor. Although some captured proteins may be false positives, the strong enrichment of PGRN binding to its putative partners, often greater than that of insulin for its receptor, together with the identification of established PGRN proteins such as sortilin, and the limited number of proteins in the PGRN capture set (33) versus the set of all glycoproteins identified (1746), suggests that the observed multiplicity of binding is an authentic reflection of how PGRN interacts with NSC-34 cells. The multiplicity of binding partners in the same cell type is consistent with reports, noted above, of multiple putative PGRN binding proteins, and suggests that PGRN may act on complexes or networks of binding proteins, rather than single dedicated receptors, as has, indeed been found for its interaction with lysosomal proteins (Paushter et al., 2018; Cui et al., 2019).

Most predicted PGRN membrane-protein partners in NSC-34 cells were located either within the plasma membrane (19/33 proteins) or the ER/Golgi/lysosome compartments (12 proteins). The enrichment of GO terms for lysosomal transport in predicted protein interaction networks supports the role of PGRN in lysosomal functions. Interestingly, however, neither the transcriptomic nor proteomic analyses detected a consistent effect of PGRN depletion on lysosomal biogenesis, even though this was strongly upregulated in PGRN knockout models (Tanaka et al., 2017). Other *in vitro* cellular systems where PGRN was depleted rather than deleted similarly displayed little transcriptional evidence for a lysosomal phenotype (Rosen et al., 2011). Additional stimuli may, therefore, be required to trigger changes in lysosome mRNA and protein expression in such cells. PGRN is a modular protein, composed of seven and a half granulin subunits, and is digested in lysosomes down to its constituent modules, which are released as granulin peptides (Salazar et al., 2015; Holler et al., 2017). This is unlikely to be a factor in the LRC experiments, however, which were conducted at 4°C, conditions that make lysosomal uptake and proteolysis unlikely. It will be interesting in future experiments to compare how the membrane interactomes of the different lysosomal granulin peptides compare to that of intact PGRN. Importantly, the lack of a strong lysosome signal in the mRNA and proteomic data suggests that PGRN exerts some biological effects independently of lysosomal protein biogenesis.

The highest scoring PGRN-binding plasma membrane protein was the tetraspanin, TSPAN4, with another tetraspanin, TSPAN31, also captured. Tetraspanins are scaffold proteins that potentiate integrin and RTK signaling by assembling molecular microdomains within the plasma membrane (Termini and Gillette, 2017), although little is known about their role in neurons. RET, the RTK signaling subunit of neurotrophic GDNF receptor, was also identified among the putative cell membrane PGRN-binding proteins, although its PGRN-binding signal (2.37-fold) was less prominent than the binding of the classic RTK ligand insulin to its receptor. PGRN expressing cells displayed enhanced RET tyrosine phosphorylation in RTK antibody arrays and Western blot assays, however, this was, at least in part, due to increased RET expression. The relative significance of PGRN interaction with RET versus the change in RET expression levels remain to be determined. Interestingly, GFRA2, an accessory subunit of the GDNF receptor that presents GDNF to RET, was recently identified as an important genetic risk factor for FTD-GRN (Pottier et al., 2018), supporting an interaction between RET and PGRN signaling. In addition to RET, NSC-34/hPGRN cells displayed enhanced phosphorylation of several other RTKs, and a similar pattern of multi-RTK activation, although for a different set of receptors, was seen in the epithelial MDA cell line. Thus, rather than target a specific receptor, high PGRN expression appears to raise the “tone” of RTK activity, that is, to mediate a general enhancement of RTK activation. The mechanisms by which PGRN achieves this remain to be defined.

Unlike RET expression, which was decreased in PGRN depleted NSC-34 cells, many other neurotrophic receptors were upregulated, including RTKs, notably the nerve growth factor receptor, and molecules in the Wnt pathway, especially FZD3, a wnt receptor family member, as well as in the notch, and semaphorin signaling pathways. The upregulation of multiple neurotrophic receptors in NSC-34/shPGRN cells may be a compensatory mechanism to maintain neurotrophic homeostasis in response to the reduced trophic support that would otherwise be supplied by PGRN as has been suggested previously for the wnt-signaling pathway (Rosen et al., 2011; Alquezar et al., 2014; de la Encarnacion et al., 2016). In support of this hypothesis, FZD3, is required for axon development in dorsal motor neurons (Hua et al., 2013). Compensatory activation of alternate receptor-based neurotrophic pathways may contribute to the surprising lack of a strong FTD-like phenotype in heterozygous *Grn*+/− mice (Filiano et al., 2013).

In conclusion, the response to PGRN expression in NSC-34 cells is complex. PGRN expression elicited cell-autonomous neurotrophic effects on cell size and axon-like extension, and supported the expression of genes for cytoskeletal regulation, and synaptic formation. This appears to be independent of pathways of lysosome biogenesis even though PGRN bound to membrane proteins in the ER/Golgi/lysosome compartments. PGRN was predicted to bind multiple cell surface proteins. The activity of RTK receptors, including RET, was increased in PGRN-rich NSC-34 cells. In conditions of PGRN insufficiency, however, the reduction in PGRN may be partially offset by enhanced expression of other neurotrophic receptors. The multiplicity of PGRN interactions with membrane proteins make it unlikely that its neurotrophic actions are mediated through a simple linear receptor signaling pathway. It may instead act through interaction with networks or complexes of membrane protein interactions. Understanding the roles of these pathways in PGRN action, and how they interrelate, and their relevance to the pathology of PGRN insufficiency will provide insights toward improved neurosupportive therapies in neurodegenerative diseases.

## Data Availability Statement

The datasets presented in this study can be found in online repositories. The names of the repository/repositories and accession number(s) can be found below: GEO, GSE185877 and ProteomeXchange, MSV000088451.

## Author Contributions

BC and AB: conceptualization, design, data curation, formal analysis, methodology, validation, and writing. BC: investigation and project administration. BC and VC-G: morphometric analysis. BC, VC-G, and AB: text review, editing and funding acquisition.

## Conflict of Interest

BC and AB have shares in Alpha Cognition Inc. This does not alter our adherence to Frontiers in Neuroscience policies on data sharing and materials. The remaining author declares that the research was conducted in the absence of any commercial or financial relationships that could be construed as a potential conflict of interest.

## Publisher’s Note

All claims expressed in this article are solely those of the authors and do not necessarily represent those of their affiliated organizations, or those of the publisher, the editors and the reviewers. Any product that may be evaluated in this article, or claim that may be made by its manufacturer, is not guaranteed or endorsed by the publisher.

## Footnotes

1
https://imagescience.org/meijering/software/neuronj/

2
https://www.uniprot.org/

3
https://www.genecards.org/

4
https://www.omim.org/
