# Supplementary material for: Multiple Molecular Pathways Are Influenced by Progranulin in a Neuronal Cell Model–A Parallel Omics Approach
Source: Front Neurosci. 2022 Jan 6;15:775391. doi: 10.3389/fnins.2021.775391 (PMC8791029; doi:10.3389/fnins.2021.775391)
Supplement: Supplementary file 1 [file Table_1.DOCX]

**Supplementary Table 1. Primers used to validate array hits.**

| S.No | Gene | Primers |
| --- | --- | --- |
| 1 | hPGRN | For 5'GGACAGTACTGAAGACTCTG’3  Rev 5'GGATGGCAGCTTGTAATGTG’3 |
| 2 | mPGRN | For 5'GCTACAGACTTAAGGAACTC’3  Rev 5'GAAATGGCAGTTTGA TACGG’3 |
| 3 | ACTIN | For 5' GAAGTGTGACGTGGACATCC’3  Rev 5' CCGATCCACACGGAGTACTT’3 |
| 4 | FGD2 | For 5’ CGAGATGAAATGGTTTCCTG’3  Rev 5’ACGGTAGTCAGAGCACTTGG’3 |
| 5 | COFILIN | For 5’ TTGTGCGGCTCCTACTAAAC’3  Rev 5’ GTCCTCACTCAGGCAAAAGA’3 |
| 7 | RHOD | For 5’AACCTGCGGAAGAAAAGACT’3  Rev 5’ TCCATCCCAGTCAACAGAGT’3 |
| 8 | STMN2 | For 5’ GAAGCTGCAGAAGAAAGACG’3  Rev 5’ GGGGTCTTTGGATTCTTTGT’3 |
| 9 | SORCS3 | For 5’ AAAGCTGCCCAAGTACTCCT’3  Rev 5’ TGGTCATCCACCTTCTTGTT’3 |
| 10 | VLDLR | For 5’TGACGCAGACTGTTCAGACC’3  Rev 5’GCCGTGGATACAGCTACCAT’3 |
| 11 | KANK1 | For 5’AGCAGACGGAGTTCTGAATG’3  Rev 5’TGCTTGCTGTCATCACTGTT’3 |
| 12 | GABRB3 | For 5’GTGCCTATCCTCGACTTTCA’3  Rev 5’ GACAAAGGCGTACTCCAGAA’3 |
| 13 | CACNG3 | For 5’ AACGAAACCAAATGGACTCA’3  Rev 5’ GAGAGTTTGTGTGGGTGGAC’3 |
| 14 | NRXN2 | For 5’AGGCCACGGTCTTAAGCTAT’3  Rev 5’ TGCCATTCATTGTCATTCAG’3 |
| 15 | SRGAP3 | For 5’ CATCGATTGCTGTGATTTGG’3  Rev 5’AGACCTGGCTGCACATATCC’3 |
| 16 | SLC32a1 | For 5’CGTCGAGGGAGACATTCATT’3  Rev 5’ATTCCTCACTGGTCGTGGTC’3 |
| 17 | SYNTAXIN 1 | For 5’ GAACAAAGTTCGCTCCAAGC’3  Rev 5’ATTCCTCACTGGTCGTGGTC’3 |
| 18 | DDC | For 5’ CCTGATTCCATTCTTTGTGG’3  Rev 5’AAGGCTCCGGTTAAGTCAGT’3 |
| 19 | DKK1 | For 5’CCCAGAAGAACCACACTGAC’3  Rev 5’CAGGTGTGGAGCCTAGAAGA’3 |
| 20 | RET | For 5’ CTCAGCATCCGCAATGGTGGT’3  Rev 5’ TGTTCTCCCTGACTCGGAAGG’3 |
| 21 | CAHT | For 5’ GGACATGATCGAGCGCTGCATC ’3  Rev 5’ GAGTCAGCTCGGACGAGCTTC ’3 |
| 22 | GAP43 | For 5’ACTGATAACTCCCCGTCCTC’3  Rev 5’GTTCAGGCATGTTCTTGGTC’3 |
| 23 | SLC18a3 | For 5’ TCTTCGACGCACTCCTACTC’3  Rev 5’ AGACGCACGGTGAGATAGAC’3 |
| 24 | NOTCH4 | For 5’ GTCACCAAGATCTGGATGAGTG ’3  Rev 5’ GTCATGGCAGGCAGCTTGGTTC ’3 |
| 25 | FZD3 | For 5’ CTACAGTGACACAAGGATCTCAC ’3  Rev 5’ CTTAATGCATCAACGTCGTAG ’3 |
| 26 | PLXNA1 | For 5’ GTCGACAAGGAAGATGGCCTG ’3  Rev 5’ GCATCCTGCCTAGAGCAGATG ’3 |
| 27 | AXL | For 5’ GAGGCCTCACCATCTCCACG ’3  Rev 5’ GAAGCCAGTGGGTCCAAGGTG ’3 |
| 28 | NGFR | For 5’ GCACCGCTGACAACCTCATTCC ’3  Rev 5’ GTCACCATTGAGCAGCTTCTC ’3 |
| 29 | CER2 | For 5’ GAAGCCAGCTGGAGATTCAC’3  Rev 5’ GACATCAGAGGCAATGCTGA’3 |
| 30 | ACAT2 | For 5’ GCTCCAGAGTGAACAGCATT’3  For 5’ TTCGTAACAGAAGCCGAATC’3 |
| 31 | CPT1B | For 5’ AAGTCATGGTGGGCAACTAA’3  Rev 5’ CTCTCCATCTGGTAGGAGCA’3 |
| 32 | HMGCR | For 5’ GGTCTGGACGAAGATGTGTC’3  Rev 5’ GCTTCTGGTTCCTTCTCACA’3 |
| 33 | NSDHL | For 5’ TCTTTATAGGCGACCTGTGC’3  Rev 5’ GGGAGGTCTTCAGTTCCATT’3 |
| 34 | LAMP1 | For 5’ GCCACTATCCAGGCCTACCTG’3  Rev 5’ CCGCAATCCACTAGATGTG’3 |
| 35 | LAMP2 | For 5’ GCTAATGGCTCAGCTTTCAAC’3  Rev 5’ TGTTGATCTGAAACGCTCTAG’3 |
| 36 | CTSD | For 5’ CACCATAAGTACAACAGTGAC’3  Rev 5’GTCCACCAGCTTCTGTTGCATC’3 |
