# Supplementary material for: Multiple Molecular Pathways Are Influenced by Progranulin in a Neuronal Cell Model–A Parallel Omics Approach
Source: Front Neurosci. 2022 Jan 6;15:775391. doi: 10.3389/fnins.2021.775391 (PMC8791029; doi:10.3389/fnins.2021.775391)
Supplement: Supplementary file 2 [file Table_2.DOCX]

**Supplementary Table 2. GO and KEGG terms upregulated in NSC-34/ shPGRN cells limited to terms downregulated with respect to both Control cells and PGRN overexpressing cells (commons). (A)** Annotation clusters of GO and KEGG terms with an enrichment score greater than one. **(B)** The top ten enriched GO terms

| 1. **DAVID Annotation clusters (enrichment score one or above) UPREGULATED in shPGRN cells vs commons in CTL and hPGRN.** | | | |
| --- | --- | --- | --- |
| UP in shPGRN v CTL and hPGRN all commons | Enrichment Score: | Number of Terms | Assignment |
|  | 2.42 | 3 | semaphorin receptor activity |
|  | 2.37 | 3 | Extracellular matrix organization |
|  | 1.55 | 4 | axon guidance |
|  | 1.42 | 6 | Extracellular matrix adhesion signaling |
|  | 1.28 | 3 | Protein glycosylation |

| 1. **Top Ten GO terms UPREGULATED in shPGRN cells vs commons in CTL and hPGRN.** | | | | | |
| --- | --- | --- | --- | --- | --- |
| Category | Term | Count | % | P-Value | Benjamini |
| GOTERM_CC_DIRECT | basement membrane | 6 | 3.8 | 6.70E-04 | 1.20E-01 |
| GOTERM_BP_DIRECT | negative regulation of RNA polymerase II regulatory region sequence-specific DNA binding | 3 | 1.9 | 8.50E-04 | 5.80E-01 |
| GOTERM_BP_DIRECT | heart development | 9 | 5.6 | 8.90E-04 | 3.70E-01 |
| GOTERM_BP_DIRECT | axon guidance | 7 | 4.4 | 1.00E-03 | 2.90E-01 |
| GOTERM_CC_DIRECT | membrane | 69 | 43.1 | 1.30E-03 | 1.20E-01 |
| GOTERM_CC_DIRECT | proteinaceous extracellular matrix | 9 | 5.6 | 2.00E-03 | 1.20E-01 |
| GOTERM_BP_DIRECT | branchiomotor neuron axon guidance | 3 | 1.9 | 3.00E-03 | 5.40E-01 |
| GOTERM_MF_DIRECT | semaphorin receptor activity | 3 | 1.9 | 4.10E-03 | 7.10E-01 |
| GOTERM_BP_DIRECT | semaphorin-plexin signaling pathway involved in axon guidance | 3 | 1.9 | 4.30E-03 | 5.80E-01 |
| GOTERM_BP_DIRECT | nerve development | 3 | 1.9 | 4.30E-03 | 5.80E-01 |
