# Supplementary figures and images for: Multiple Molecular Pathways Are Influenced by Progranulin in a Neuronal Cell Model–A Parallel Omics Approach
Source: Front Neurosci. 2022 Jan 6;15:775391. doi: 10.3389/fnins.2021.775391 (PMC8791029; doi:10.3389/fnins.2021.775391)

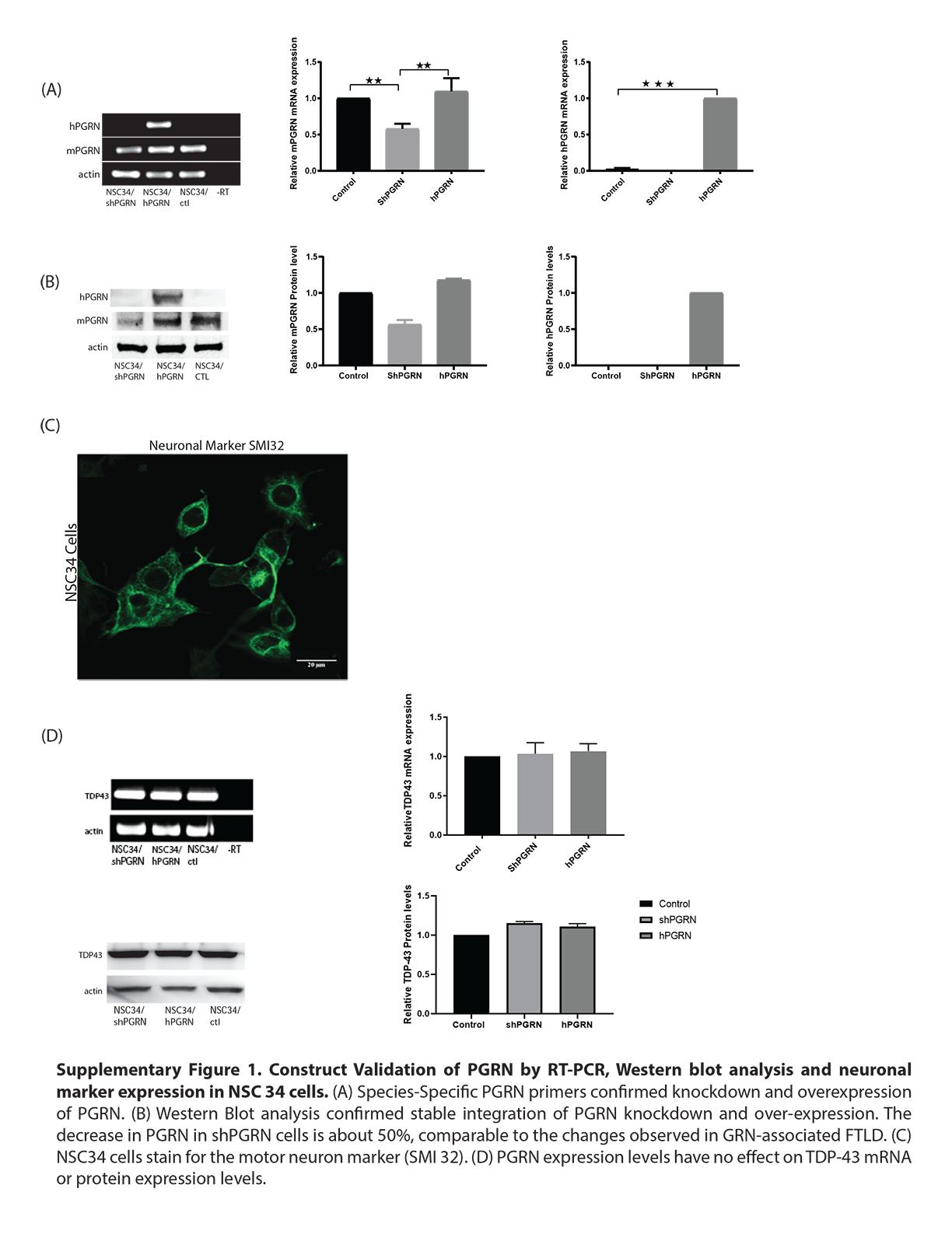

Supplement: Supplementary file 4 [file Image_1.TIF]

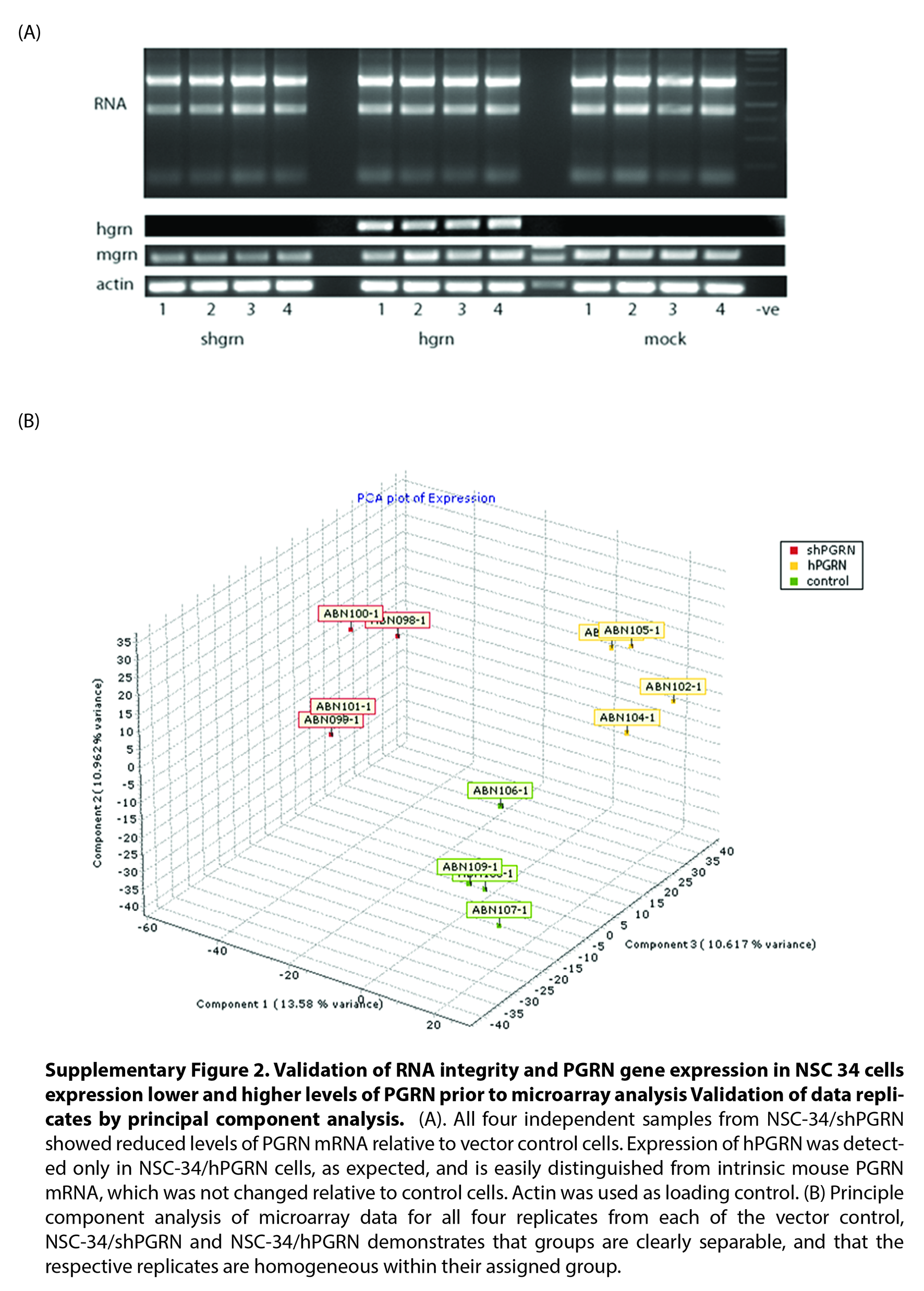

Supplement: Supplementary file 5 [file Image_2.TIF]

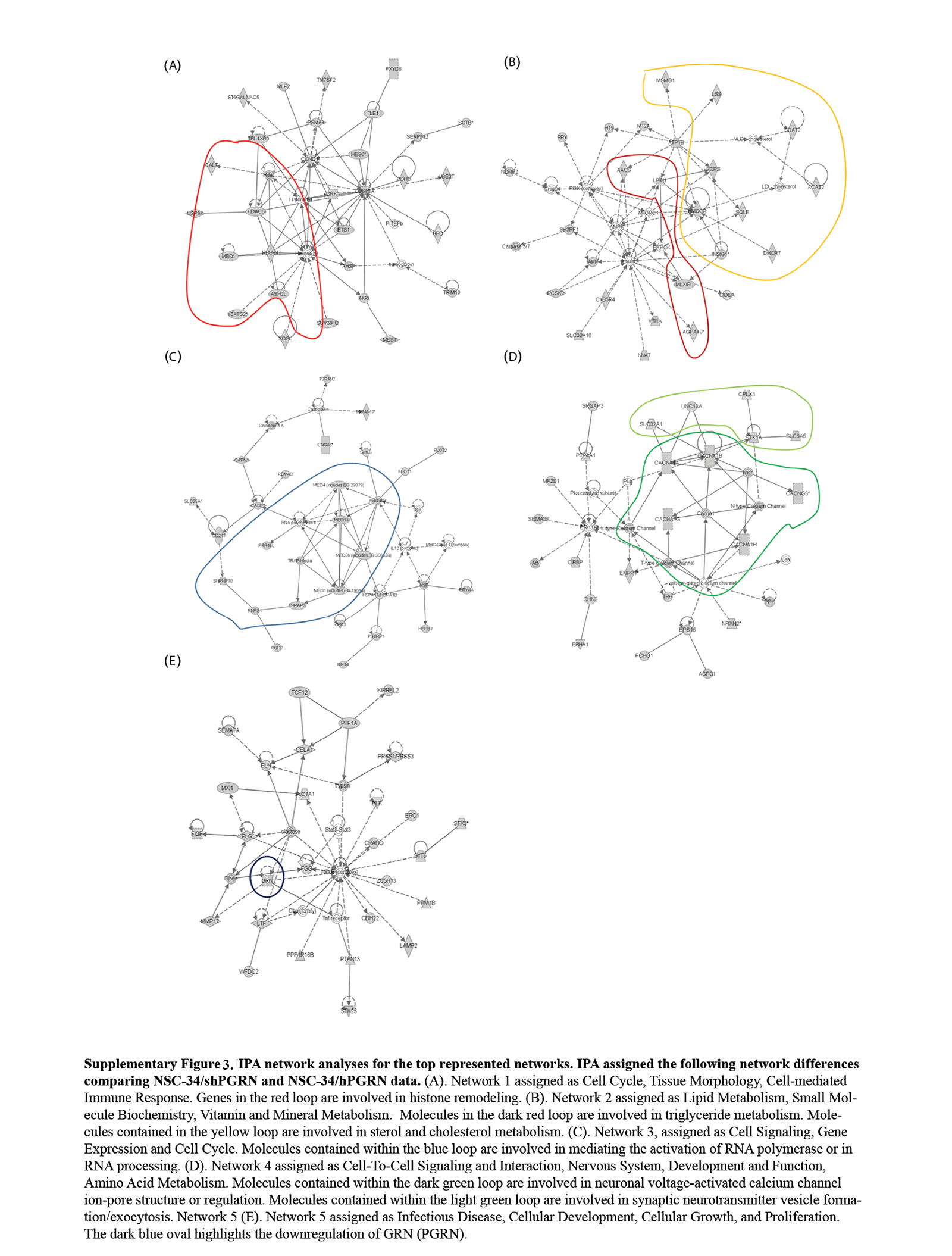

Supplement: Supplementary file 6 [file Image_3.TIF]

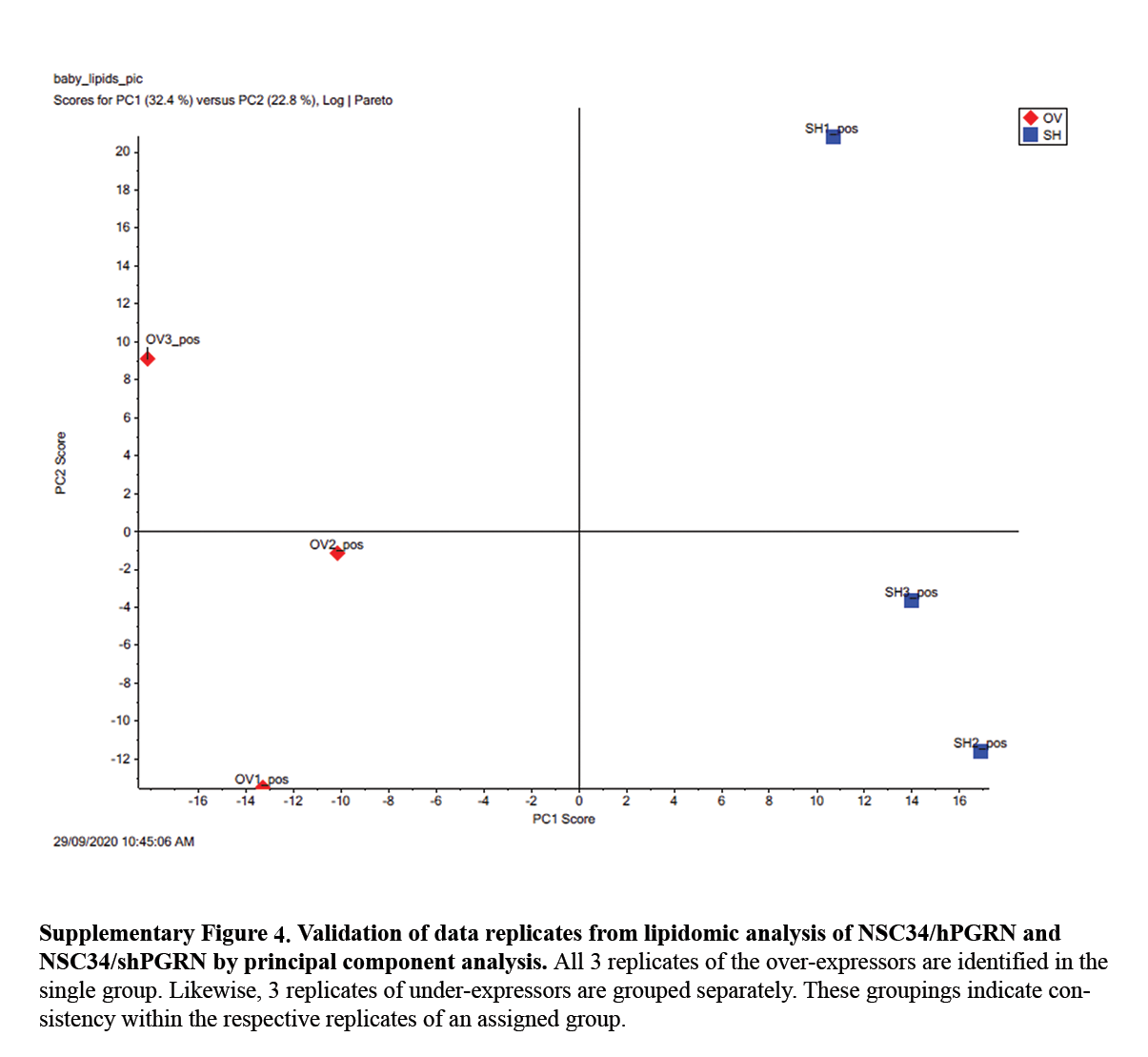

Supplement: Supplementary file 7 [file Image_4.TIF]

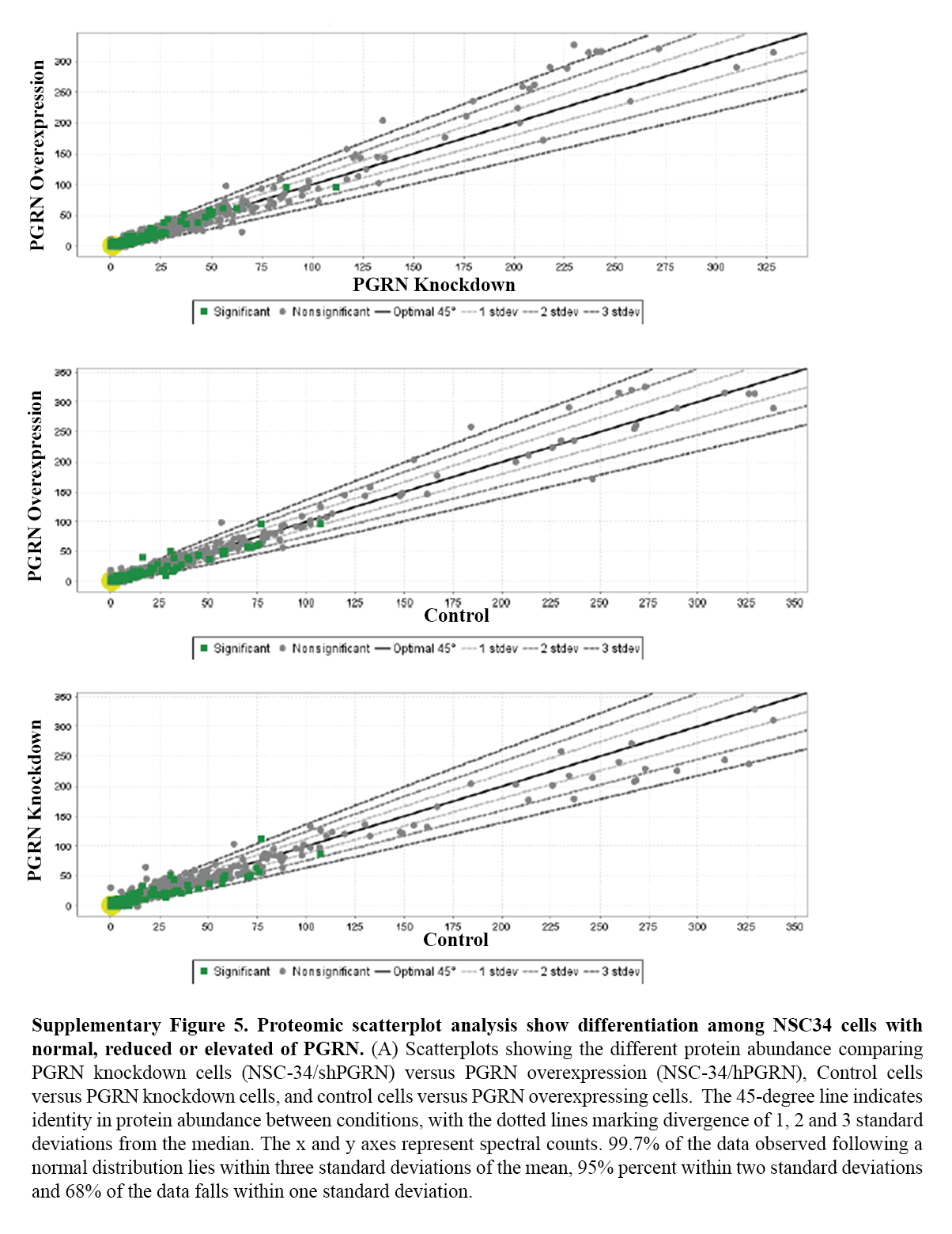

Supplement: Supplementary file 8 [file Image_5.TIF]
